# P-Rex2 suppresses glucose uptake into liver and skeletal muscle through different adaptor functions

**DOI:** 10.1038/s41598-025-01720-w

**Published:** 2025-08-05

**Authors:** Elpida Tsonou, Julia Y. Chu, Polly A. Machin, Anna G. Roberts, Anne Segonds-Pichon, David Baker, David C. Hornigold, Heidi C. E. Welch

**Affiliations:** 1 Signalling Programme, The Babraham Institute, Babraham Research Campus, Cambridge, CB22 3AT UK; 2 Bioscience Metabolism, Research and Early Development, Cardiovascular, Renal and Metabolism (CVRM), BioPharmaceuticals R&D, AstraZeneca, Cambridge, UK; 3 Bioinformatics Facility, The Babraham Institute, Cambridge, UK

**Keywords:** P-Rex2, P-Rex1, Guanine-nucleotide exchange factor (GEF), Glucose homeostasis, Liver, Skeletal muscle, Mitochondria, G protein-coupled receptor (GPCR), Gpr21, RHO signalling, Phosphoinositol signalling, Metabolism, Type 2 diabetes

## Abstract

P-Rex2 is a Rac guanine-nucleotide factor (Rac-GEF) that controls glucose homeostasis. This role is thought to be mediated through its adaptor function inhibiting Pten rather than through its Rac-GEF activity, but this remains to be demonstrated. To examine this question, we have investigated the roles of P-Rex2 in glucose homeostasis using *Prex2*^*–/–*^ and catalytically-inactive *Prex2*^*GD*^ mice. We show that P-Rex2 is required for insulin sensitivity but limits glucose clearance, suppressing glucose uptake into liver and skeletal muscle independently of its catalytic activity. In hepatocytes, P-Rex2 suppresses Glut2 cell surface levels, mitochondrial membrane potential and mitochondrial ATP production. We identify the orphan GPCR Gpr21 as a P-Rex2 target and propose that P-Rex2 limits hepatic glucose clearance by controlling Gpr21 trafficking. In skeletal muscle cells, P-Rex2 suppresses glucose uptake through a separate adaptor function, independently of Gpr21. Additionally, P-Rex2 suppresses insulin secretion by pancreatic islets and plasma insulin levels. Finally, P-Rex2 plays distinct Rac-GEF activity dependent and independent roles in PIP_3_ production in liver and skeletal muscle, respectively. Together, our study identifies complex roles of P-Rex2 in glucose homeostasis, mediated through largely GEF-activity independent mechanisms which include the GPCR Gpr21 in hepatocytes and but are not obviously linked to the regulation of Pten.

## Introduction

Glucose homeostasis is the maintenance of blood glucose levels within their physiological range. A rise in blood glucose level following a meal stimulates glucose clearance into the brain and liver independently of insulin signalling, and glucose clearance into adipose and skeletal muscle tissues in an insulin-dependent manner. A drop in blood glucose level during fasting stimulates the release of glucose into the blood stream through glycogenolysis and gluconeogenesis in the liver. Chronic hyperglycaemia can lead to metabolic syndrome, which comprises obesity, insulin resistance, type 2 diabetes, and increased risk of other pathologies including cardiovascular disease and cancer^1^.

Ras-related C3 botulinum toxin substrate 1 (Rac1) is a small GTPase (guanine nucleotide-binding protein) that is increasingly recognised as a regulator of glucose homeostasis, largely through its role in remodelling actin cytoskeletal structure to support secretory processes^2,3^. In skeletal muscle, Rac1 mediates the insulin- or exercise-stimulated translocation of the glucose transporter Glut4 to the plasma membrane and glucose uptake^4–7^. In pancreatic β cells, Rac1 controls glucose-stimulated insulin release^8,9^. Mice with skeletal-muscle specific Rac1 deficiency are glucose intolerant and insulin resistant^6^. Rac1 is activated by numerous guanine-nucleotide exchange factors (Rac-GEFs), several of which have been implicated in the control of glucose homeostasis^2^. Currently best-understood are Rac-GEFs from the Vav family. Mice with a mutation that reduces the activity of Vav2 exhibit low skeletal muscle mass, hyperglycaemia, glucose intolerance, increased adiposity, and liver steatosis^10^, whereas mice with hyperactive Vav2 show increased muscle mass and resistance to developing metabolic syndrome^10–12^. Mice deficient in Vav3 develop metabolic function-associated fatty liver disease (MAFLD) and type 2 diabetes when fed a normal chow diet but are protected from obesity and metabolic syndrome when on a high-fat diet (HFD), due to increased energy consumption and brown adipose tissue thermogenesis through diet-induced chronic excitation of the sympathetic nervous system^13^.

Phosphatidylinositol-3,4,5-trisphosphate dependent Rac exchanger 2 (P-Rex2) is a widely expressed Rac-GEF that makes up the P-Rex family together with P-Rex1^14–18^. P-Rex1 and P-Rex2 have a unique synergistic mode of activation by the lipid second messenger phosphatidylinositol-3,4,5-trisphosphate (PIP_3_), which is generated by phosphoinositide 3-kinase (PI3K), and by the Gβγ subunits of heterotrimeric G proteins, making these GEFs ideal mediators of G protein-coupled receptor (GPCR) signalling^17^. P-Rex2 is mostly known for its pathological roles in cancer. Unusually for Rac-GEFs, the *PREX2* gene is frequently mutated in human cancers, particularly melanoma and pancreatic cancer^19,20^, and mutations are associated with melanoma tumorigenesis^19,21,22^ and tumour cell invasiveness^23^. Relatively little is known about physiological roles of P-Rex2. The Rac-GEF regulates motor coordination^15^, synaptic plasticity in cerebellar Purkinje neurons^24^, lipogenesis in sebocytes^25^, and mechanosensing in endothelial cells^26^. Importantly, P-Rex2 also controls glucose homeostasis^2^. *Prex2*^*–/–*^ mice are reportedly glucose intolerant and insulin resistant^27^. In addition to being a target of the PI3K/PIP_3_ signalling pathway, P-Rex2 also inhibits the tumour suppressor phosphatase and tensin homolog deleted on chromosome 10 (Pten) which dephosphorylates PIP_3_, through a Rac-GEF activity independent adaptor function, thereby increasing PI3K signalling pathway activity^28^. It is thought that P-Rex2 controls glucose homeostasis through this Pten inhibition rather than through its Rac-GEF activity^27^, but that remains to be demonstrated.

The P-Rex2 homologue P-Rex1 has been studied in more detail than P-Rex2, and several roles in glucose homeostasis have been identified. In 3T3-L1 adipocytes, P-Rex1 is required for the insulin-stimulated translocation of Glut4 from storage vesicles to the plasma membrane, promoting insulin-stimulated glucose uptake in a Rac-GEF activity dependent manner^29^. In brown adipocytes, P-Rex1 regulates thermogenic potential^30^. Adenoviral P-Rex1 depletion protects mice from HFD-induced MAFLD^31^. We recently investigated the role of P-Rex1 in glucose homeostasis using *Prex1*^*–/–*^ and catalytically-inactive *Prex1*^*GD*^ mice^32^. We showed that P-Rex1 maintains fasting blood glucose levels and insulin sensitivity through its Rac-GEF activity but limits glucose clearance independently of its catalytic activity, throughout ageing. *Prex1*^*–/–*^ mice were protected from developing HFD-induced diabetes. The increased glucose clearance in *Prex1*^*–/–*^ mice stemmed, at least in part, from constitutively enhanced hepatic glucose uptake. P-Rex1 limits the upregulation of Glut2, mitochondrial membrane potential and ATP production in hepatocytes, and controls mitochondrial morphology, independently of its catalytic activity. We identified the inhibitory orphan GPCR Gpr21 as a target of P-Rex1 in these processes^32^.

P-Rex2 and P-Rex1 have in common their domain structure and their regulation by PIP_3_ and Gβγ^17^. As targets of PI3K, both GEFs mediate insulin signalling^27,28,33^, and both interact with mammalian target of rapamycin (mTOR), a central controller of cell growth and metabolism^34^. In addition, we recently showed that both regulate GPCR trafficking, limiting the agonist-induced internalisation of active GPCRs through an adaptor function that involves binding to G protein-coupled receptor kinase 2 (Grk2) and the regulation of GPCR phosphorylation^35^. We showed that P-Rex1 controls the trafficking of the inhibitory orphan GPCR Gpr21 and proposed that this underlies its function in hepatic glucose uptake^32^. Unlike P-Rex2, however, P-Rex1 cannot bind and inhibit Pten^28^, and therefore the mechanisms through which these Rac-GEFs control glucose metabolism are likely to differ.

In-light-of our recent findings on P-Rex1, we revisited here the role of P-Rex2 in glucose homeostasis. We show that P-Rex2 is required for insulin sensitivity, as was expected, but in contrast to expectations suppresses glucose clearance rather than promoting it. Like P-Rex1, P-Rex2 limits glucose uptake in liver cells through an adaptor function involving Gpr21. However, unlike P-Rex1, P-Rex2 also limits glucose uptake in skeletal muscle cells, independently of Gpr21. Furthermore, P-Rex2 limits glucose-stimulated plasma insulin levels and insulin secretion by pancreatic islets. In addition, P-Rex2 has Rac-GEF activity dependent and independent roles in insulin-stimulated PIP_3_ production. Thus, our study identifies complex, unexpected adaptor functions of P-Rex2 in glucose homeostasis, only one of which, the insulin-stimulated production of PIP_3_ in skeletal muscle, can comfortably be linked to the inhibition of Pten.

## Results

### P-Rex2 limits glucose tolerance and is required for insulin sensitivity, independently of its catalytic activity

To study the role of P-Rex2 in glucose homeostasis, we tested the fasting blood glucose levels, glucose tolerance and insulin sensitivity of *Prex2*^*–/–*^ mice^15^ throughout ageing from young adult (10 weeks) to post-reproductive ‘young-old’ age (1 year), equivalent to 20 and 60 years in humans^36^, respectively. To assess if any roles of P-Rex2 in glucose homeostasis require its catalytic Rac-GEF activity, we generated a GEF-dead P-Rex2 mouse strain, *Prex2*^*GD*^, using CRISPR/Cas9 gene editing to introduce two mutations, E22A and N204A, in the catalytic Dbl homology (DH) domain which render the protein catalytically inactive^32,37,38^ (Supplementary Figure S1A). *Prex2*^*GD*^ mice were viable, fertile, and showed no overt phenotype. The introduction of the E22A and N204A mutations did not affect the expression level of the P-Rex2 protein (Supplementary Figure S1B).

**Fig. 1:**
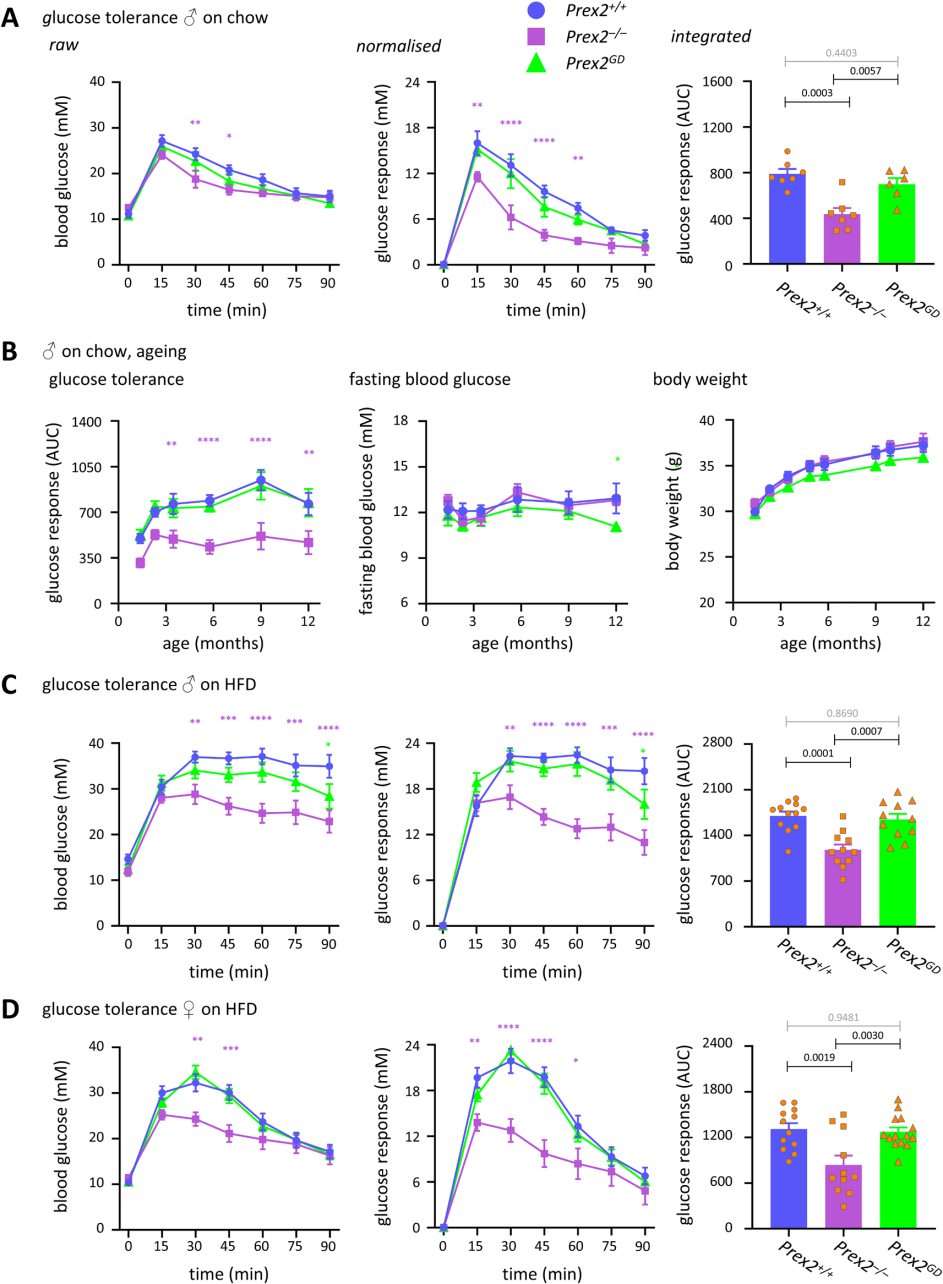
P-Rex2 limits glucose clearance in mice throughout ageing, independently of its GEF-activity, contributing to diet-induced glucose intolerance. (**A, B**) Glucose tolerance on chow diet. (**A**) The glucose tolerance of male *Prex2*^+*/*+^ (blue, circles), *Prex2*^*–/–*^ (purple, squares), and *Prex2*^*GD*^ (green, triangles) mice on chow diet was measured at the age of 6 months. Food was withdrawn for 6 h, fasting blood glucose tested, 2 g/kg glucose injected *i.p.,* and blood glucose assessed again at the indicated time points. Left-hand panel: blood glucose concentration, middle: glucose response normalised to fasting blood glucose, right: integrated glucose response (AUC). Data are mean ± SEM of mice pooled from 2 independent cohorts of 3–4 mice per genotype; beige dots show individual mice. (**B**) Glucose tolerance (integrated response), fasting blood glucose and body weight of the cohorts in (**A**) measured at the indicated ages. (**C, D**) Glucose tolerance on high-fat diet (HFD). The glucose tolerance of 6-month-old male (**C**) and female (**D**) *Prex2*^+*/*+^, *Prex2*^*–/–*^, and *Prex2*^*GD*^ mice on HFD was measured as in (**A**). Data are mean ± SEM of 3 independent cohorts of 3–4 (**C**) or 3–5 (**D**) mice per genotype; beige dots show individual mice. Statistics in time courses are two-way ANOVA with Sidak’s multiple comparisons correction; purple stars denote significance between *Prex2*^+*/*+^ and *Prex2*^*–/–*^, green between *Prex2*^+*/*+^ and *Prex2*^*GD*^. Statistics in bar graphs are one-way ANOVA with Tukey’s multiple comparisons test; *p*-values in black denote significant differences, *p*-values in grey are non-significant.

*Prex2*^*–/–*^ mice showed improved glucose tolerance compared to wild type, whereas *Prex2*^*GD*^ mice did not (Fig. 1A). Hence, P-Rex2 limits glucose tolerance, and it does so through an adaptor function rather than through its catalytic Rac-GEF activity. The enhanced glucose tolerance of *Prex2*^*–/–*^ mice persisted throughout ageing (Fig. 1B), despite normal body and organ weights, and normal fasting blood glucose levels throughout ageing (Fig. 1B, Supplementary Figure S2). Furthermore, both male and female *Prex2*^*–/–*^ mice on high-fat diet (HFD) were protected from developing diet-related glucose intolerance, the males partially and the females fully (Fig. 1C, D). This was seen despite normal fasting glucose levels in *Prex2*^*–/–*^ mice on HFD, and despite *Prex2*^*–/–*^ females gaining diet-related weight more rapidly than *Prex2*^+*/*+^ or *Prex2*^*GD*^ females (Supplementary Figure S1C-D). In contrast to *Prex2*^*–/–*^, the glucose tolerance of *Prex2*^*GD*^ mice on HFD was normal. Therefore, P-Rex2 limits glucose tolerance independently of its Rac-GEF activity, throughout ageing and regardless of sex and diet, rather than promoting glucose tolerance as would have been expected^27^.

**Fig. 2:**
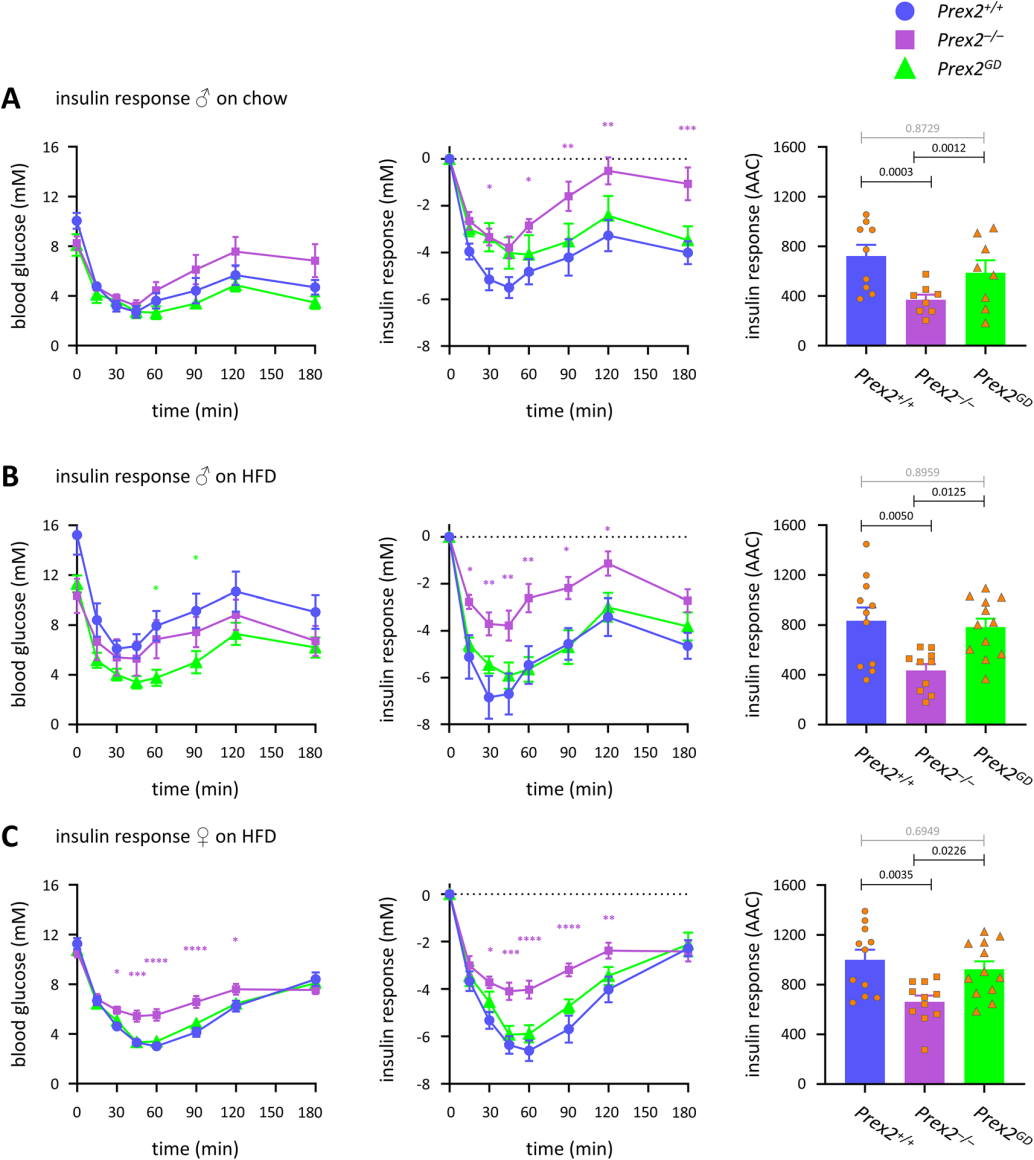
P-Rex2 is required for insulin sensitivity, independently of its catalytic activity. (**A–C**) Insulin sensitivity. *Prex2*^+*/*+^ (blue, circles), *Prex2*^*–/–*^ (purple, squares), and *Prex2*^*GD*^ (green, triangles) mice were fasted for 4 h, fasting blood glucose was measured, 0.75 IU/kg insulin *s.c.* injected, and blood glucose tested at the indicated timepoints. Mice in (**A**) were 10-month-old males on chow diet, in (**B**, **C**) 5-month-old males (**B**) or females (**C**) on HFD. Data are mean ± SEM of mice pooled from 2 independent cohorts of 3–4 mice per genotype (**A**), or 3 cohorts of 3–4 (**B**) or 3–5 (**C**) mice per genotype, the same cohorts as in Fig. 1; beige dots show individual mice. Left-hand panels: blood glucose concentration, middle: insulin response normalised to fasting blood glucose, right: integrated insulin response (AAC), beige dots show individual mice. Statistics in time courses are two-way ANOVA with Sidak’s multiple comparisons correction; purple stars denote significance between *Prex2*^+*/*+^ and *Prex2*^*–/–*^. Statistics in bar graphs are one-way ANOVA with Tukey’s multiple comparisons test; *p*-values in black denote significant differences, *p*-values in grey are non-significant.

*Prex2*^*–/–*^ mice were insulin resistant. This was seen in old *Prex2*^*–/–*^ mice on chow diet, and in middle-aged *Prex2*^*–/–*^ mice on HFD, both males and females. In contrast, the insulin sensitivity of P*rex2*^*GD*^ mice was normal (Fig. 2). Hence, P-Rex2 is required for insulin sensitivity, as expected from a previous report^27^, and it exerts this role through an adaptor function which is independent of sex and diet.

### P-Rex2 limits glucose-stimulated plasma insulin levels and pancreatic insulin secretion, independently of its catalytic activity

**Fig. 3:**
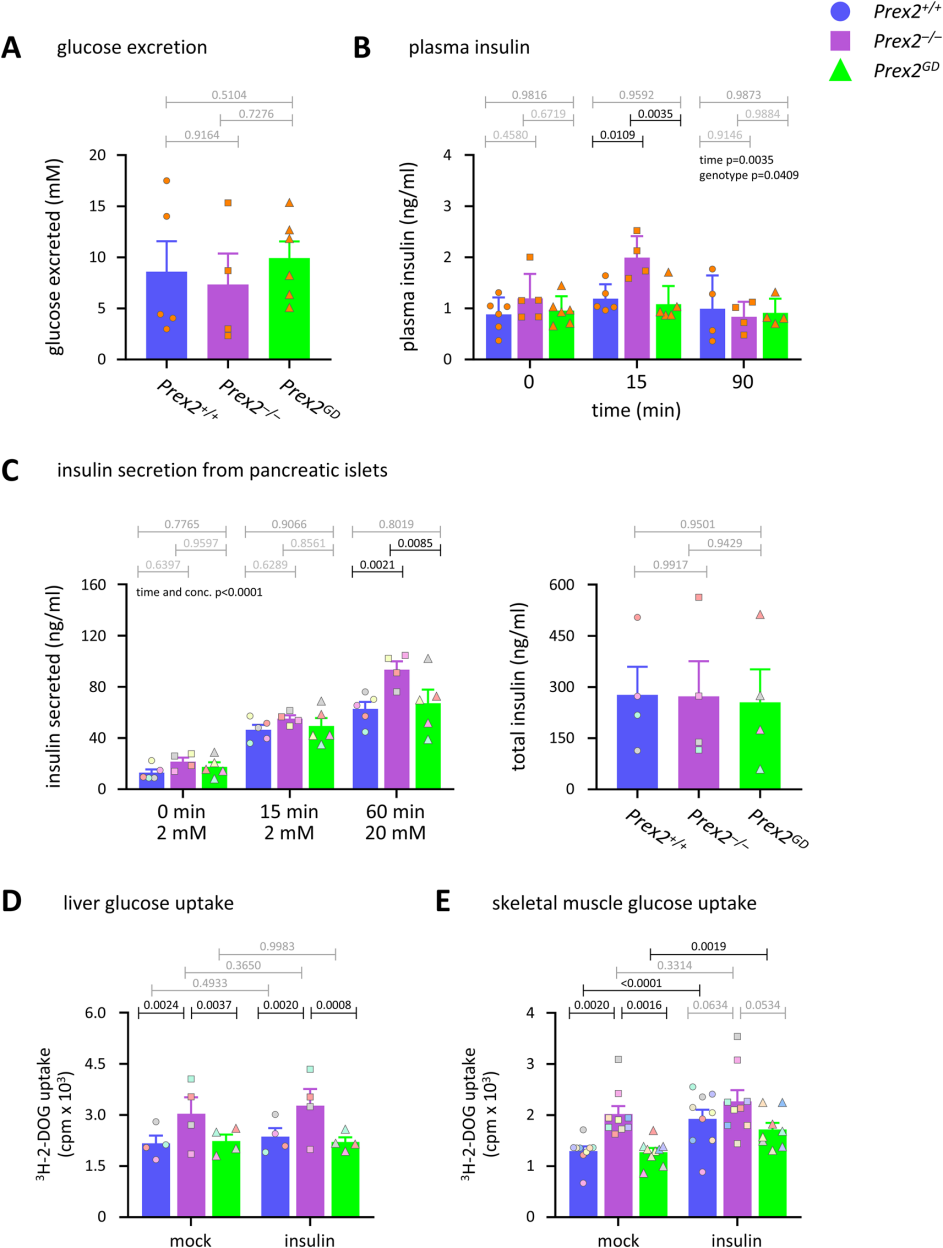
P-Rex2 limits glucose-stimulated plasma insulin, pancreatic insulin secretion, and glucose uptake into liver and skeletal muscle, independently of its catalytic activity. (**A**) Glucose excretion. 15-week-old male and female *Prex2*^+*/*+^ (blue, circles), *Prex2*^*–/–*^ (purple, squares), and *Prex2*^*GD*^ (green, triangles) mice on HFD were fasted for 6 h before 2 g/kg glucose was *i.p.* injected*.* Urine was collected 90 min later and glucose concentration in urine assessed by ELISA. Data are mean ± SEM of 5 *Prex2*^+*/*+^, 4 *Prex2*^*–/–*^, and 6 *Prex2*^*GD*^ mice pooled from 2 independent cohorts or 2–3 mice per genotype; beige symbols show individual mice. Statistics are one-way ANOVA with Tukey’s multiple comparisons test; *p*-values in grey are non-significant. (**B**) Plasma insulin. 15-week-old male *Prex2*^+*/*+^, *Prex2*^*–/–*^, and *Prex2*^*GD*^ mice on HFD were fasted for 6 h and injected *i.p.* with water (0 time) or 2 g/kg glucose for 15 or 90 min, and insulin in blood plasma was analysed by ELISA. Data are mean ± SEM of mice pooled from 4 independent cohorts of 1–2 mice per group; beige symbols show individual mice. Statistics are two-way ANOVA with Sidak’s multiple comparisons correction. (**C**) Glucose-stimulated insulin secretion from pancreatic islets. Islets of Langerhans were isolated from the pancreas of 16-week-old female *Prex2*^+*/*+^, *Prex2*^*–/–*^, and *Prex2*^*GD*^ mice on HFD. Left: Insulin secreted by 10 islets before and after stimulation with 2 mM glucose for 15 min and then 20 mM glucose for a further 45 min was detected by ELISA. Right: total insulin in lysates of 5 pancreatic islets. Data are mean ± SEM of 4–5 independent experiments; symbol colours mark individual experiments. Statistics on left are two-way ANOVA with Sidak’s multiple comparisons corrections, on right one-way ANOVA with Tukey’s multiple comparisons. (**D, E**) Glucose uptake into (D) liver cells and (E) skeletal muscle cells. Liver and skeletal muscle cells from 15-week-old *Prex2*^+*/*+^, *Prex2*^*–/–*^, and *Prex2*^*GD*^ mice on chow diet were stimulated with 100 nM insulin for 10 min at 37 °C, or mock-stimulated, followed by the addition of 50 μM 2-DOG, 0.25 μCi ^3^H-2-DOG for 60 min. Cells were washed, lysed, and glucose uptake was measured by scintillation counting. Data are mean ± SEM of (**D**) 4 and (**E**) 9 independent experiments; symbol colours mark individual experiments. Statistics are two-way ANOVA with Sidak’s multiple comparisons correction. (**A**–**E**) *P*-values in black denote significant differences, *p*-values in grey are non-significant.

To investigate the improved glucose tolerance of *Prex2*^*–/–*^ mice, we evaluated these mice in metabolic cages, where they performed normally regardless of age, sex, and diet (Supplementary Figure S3). We asked whether *Prex2*^*–/–*^ mice can clear glucose more efficiently through increased excretion, but glucose excretion in urine was normal (Fig. 3A). These results implied that the increased glucose tolerance of *Prex2*^*–/–*^ mice is caused by enhanced glucose clearance into tissues.

We assessed if *Prex2*^*–/–*^ mice might clear glucose better because of increased insulin levels. Plasma insulin levels were normal in fasted *Prex2*^*–/–*^ mice but transiently increased in *Prex2*^*–/–*^ mice upon glucose challenge more than in wild type (Fig. 3B). This elevated glucose-stimulated plasma insulin may contribute to the increased glucose clearance in *Prex2*^*–/–*^ mice. In contrast to *Prex2*^*–/–*^, plasma insulin was normal in P*rex2*^*GD*^ mice (Fig. 3B), suggesting that P-Rex2 regulates insulin levels through an adaptor function. In line with the raised plasma insulin, the glucose-stimulated secretion of insulin was raised in pancreatic islets isolated from *Prex2*^*–/–*^ mice compared to wild type, although only after prolonged stimulation with a high concentration of glucose, whereas insulin secretion from P*rex2*^*GD*^ islets was normal (Fig. 3C). Therefore, P-Rex2 limits glucose-stimulated plasma insulin levels and pancreatic insulin secretion through an adaptor function.

### P-Rex2 limits glucose uptake into liver and skeletal muscle cells, independently of its catalytic activity

We sought to identify the *Prex2*^*–/–*^ tissues which show enhanced glucose clearance. Histological analysis of metabolic tissues revealed a tendency towards elevated glycogen storage in the liver of *Prex2*^*–/–*^ mice. However, this was seen only in middle-aged animals and remained within the normal range (Supplementary Figure S4A), so it seems unlikely that increased hepatic glycogen storage is a key explanation for the increased glucose clearance in *Prex2*^*–/–*^ mice. The histological analysis of skeletal muscle and of white and brown adipose tissues revealed no abnormalities (Supplementary Figure S4B-D). To measure glucose uptake, we determined the constitutive and insulin-stimulated uptake of tritiated 2-deoxyglucose (^3^H-2-DOG) into isolated liver, skeletal muscle, and mature adipose cells from *Prex2*^+*/*+^, *Prex2*^*–/–*^ and *Prex2*^*GD*^ mice. *Prex2*^*–/–*^ liver cells and skeletal muscle cells showed constitutively increased glucose uptake, whereas glucose uptake was normal in *Prex2*^*GD*^ cells (Fig. 3D, E). Thus, P-Rex2 limits constitutive glucose uptake into liver and skeletal muscle cells, independently of its Rac-GEF activity. In the liver, insulin stimulation did not affect glucose uptake, as expected^39,40^. In skeletal muscle cells, insulin stimulation increased glucose uptake in P*rex2*^+*/*+^ and *Prex2*^*GD*^ cells but did not increase further the constitutively raised response of *Prex2*^*–/–*^ cells (Fig. 3D, E). In contrast to liver and skeletal muscle, glucose uptake into mature *Prex2*^*–/–*^ visceral white adipocytes, subcutaneous white adipocytes, and interscapular brown adipocytes was normal. Insulin stimulation raised glucose uptake in all types of adipocytes, but to a similar level in all genotypes, except for a slight increase in visceral white *Prex2*^*–/–*^ adipocytes (Supplementary Figure S5). Therefore, P-Rex2 suppresses glucose uptake in liver and skeletal muscle cells through an adaptor function but does not control glucose uptake in adipose cells. The raised glucose uptake into liver and skeletal muscle cells can explain the increased glucose tolerance of *Prex2*^*–/–*^ mice. As enhanced glucose uptake into these organs occurred constitutively, independently of insulin, the raised pancreatic insulin secretion and plasma insulin seem unlikely to be major contributors to the increased glucose clearance in *Prex2*^*–/–*^ mice.

### P-Rex2 suppresses Glut2 surface level, mitochondrial membrane potential, and mitochondrial ATP production in liver cells

**Fig. 4:**
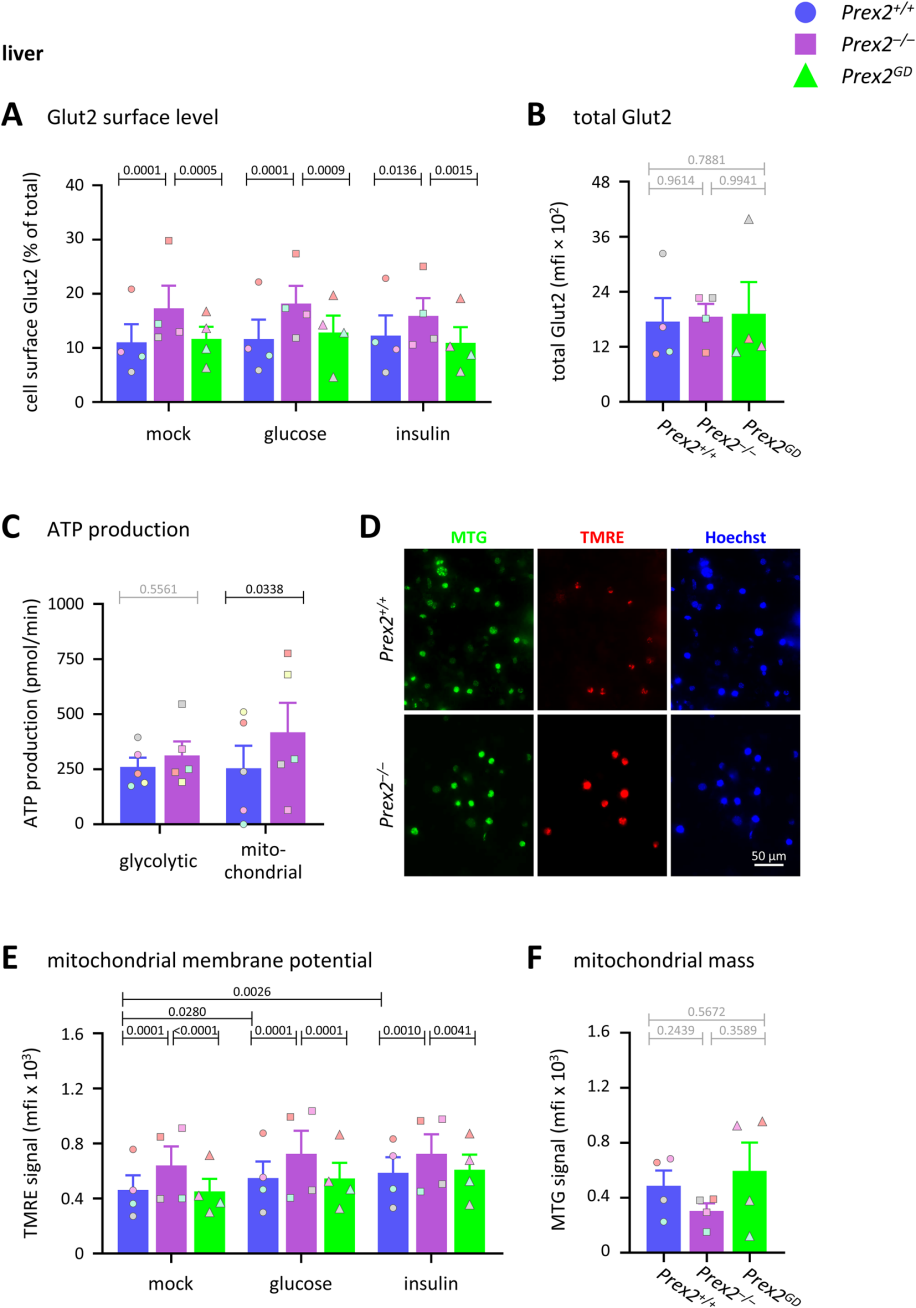
P-Rex2 limits Glut2 surface level, mitochondrial membrane potential, and mitochondrial ATP production in liver cells. (**A**) Glut2 cell surface level. Liver cells from 15-week-old *Prex2*^+*/*+^ (blue, circles), *Prex2*^*–/–*^ (purple, squares), and *Prex2*^*GD*^ (green, triangles) mice on chow diet were stimulated with 5 mM glucose or 100 nM insulin for 10 min at 37 °C, or were mock-stimulated, stained with Glut2 antibody, analysed by flow cytometry, and the mean fluorescence intensity (mfi) of Glut2 surface level expressed as % of total Glut2. Data are mean ± SEM of 4 independent experiments; symbol colours mark individual experiments. (**B**) Total Glut2 was measured as in (A) except in permeabilised cells. Data are mean ± SEM of 4 independent experiments; beige symbols show individual experiments. (**C**) ATP production. Liver cells were analysed by Seahorse assay to quantify constitutive ATP production from glycolytic and mitochondrial respiration. Data are mean ± SEM of 5 independent experiments; symbol colours mark individual experiments. (**D–F**) Mitochondrial membrane potential. Liver cells were stained with MitoTracker Green (MTG), TMRE, and Hoechst 33342 DNA dye, and analysed by immunofluorescence microscopy (**D**), or they were stimulated with 5 mM glucose or 100 nM insulin for 30 min at 37 °C, or mock-treated, before staining with MTG and TMRE, and analysis by flow cytometry for (**E**) TMRE signal in MTG^+^ cells to quantify mitochondrial membrane potential and (**F**) MTG signal to quantify mitochondrial mass. Images in (**D**) are representative of 3 independent experiments. Data in (**E**, **F**) are mean ± SEM of 4 independent experiments; symbol colours mark individual experiments. Statistics in (**A**, **C**, **E**) are two-way ANOVA with Sidak’s multiple comparisons correction. Statistics in (**B**, **F**) are one-way ANOVA with Tukey’s multiple comparisons correction. *P*-values in black denote significant differences, *p*-values in grey are non-significant.

Glucose enters liver cells mainly through the glucose transporter Glut2, which is sensitive to glucose but not insulin^39,40^. We measured the total and cell surface levels of Glut2 by flow cytometry. Cell surface Glut2 was constitutively raised in *Prex2*^*–/–*^ liver cells, independently of stimulation with glucose or insulin, whereas it was normal in *Prex2*^*GD*^ liver cells (Fig. 4A). These data suggest that P-Rex2 suppresses hepatic glucose uptake by limiting the cell surface localisation of Glut2, independently of its catalytic activity. The total cellular level of Glut2 was normal in all genotypes (Fig. 4B), suggesting that P-Rex2 might regulate Glut2 trafficking.

To investigate if *Prex2*^*–/–*^ liver cells metabolise glucose more readily, we measured glycolytic and mitochondrial ATP production by Seahorse assay. *Prex2*^*–/–*^ liver cells showed constitutively increased mitochondrial ATP production, whereas glycolytic ATP production was normal (Fig. 4C and Supplementary Figure S6A). Therefore, P-Rex2 suppresses mitochondrial ATP production.

To investigate if P-Rex2 controls mitochondrial ATP production by affecting mitochondrial membrane potential, we stained liver cells from *Prex2*^+*/*+^, *Prex2*^*–/–*^ and *Prex2*^*GD*^ mice with MitoTracker Green (MTG) to quantify mitochondrial mass and with tetramethyl-rhodamine ethyl ester (TMRE) to report mitochondrial membrane potential, and we analysed the cells by immunofluorescence microscopy and flow cytometry. *Prex2*^*–/–*^ liver cells but not *Prex2*^*GD*^ cells showed constitutively increased TMRE signal, which remained elevated after glucose or insulin stimulation (Fig. 4D, E). In contrast, the MTG signal was normal (Fig. 4D, F). Hence, P-Rex2 suppresses mitochondrial membrane potential in liver cells, independently of its catalytic activity, without affecting mitochondrial mass.

### P-Rex2 suppresses glucose uptake into liver cells through Gpr21

Together, data in Figs. 3 and 4 showed that P-Rex2 suppresses hepatic glucose uptake, Glut2 trafficking, mitochondrial membrane potential, and mitochondrial glucose metabolism to ATP. We recently showed that the P-Rex2 homologue P-Rex1 plays similar roles in the liver, through an adaptor function involving the trafficking of the constitutively-active orphan GPCR Gpr21, a receptor which inhibits glucose uptake into liver cells^32^. Furthermore, we also recently showed that both P-Rex1 and P-Rex2 control the trafficking of active GPCRs^35^. Therefore, by analogy to P-Rex1, we hypothesized that P-Rex2 might suppress hepatic glucose metabolism by controlling Gpr21.

**Fig. 5:**
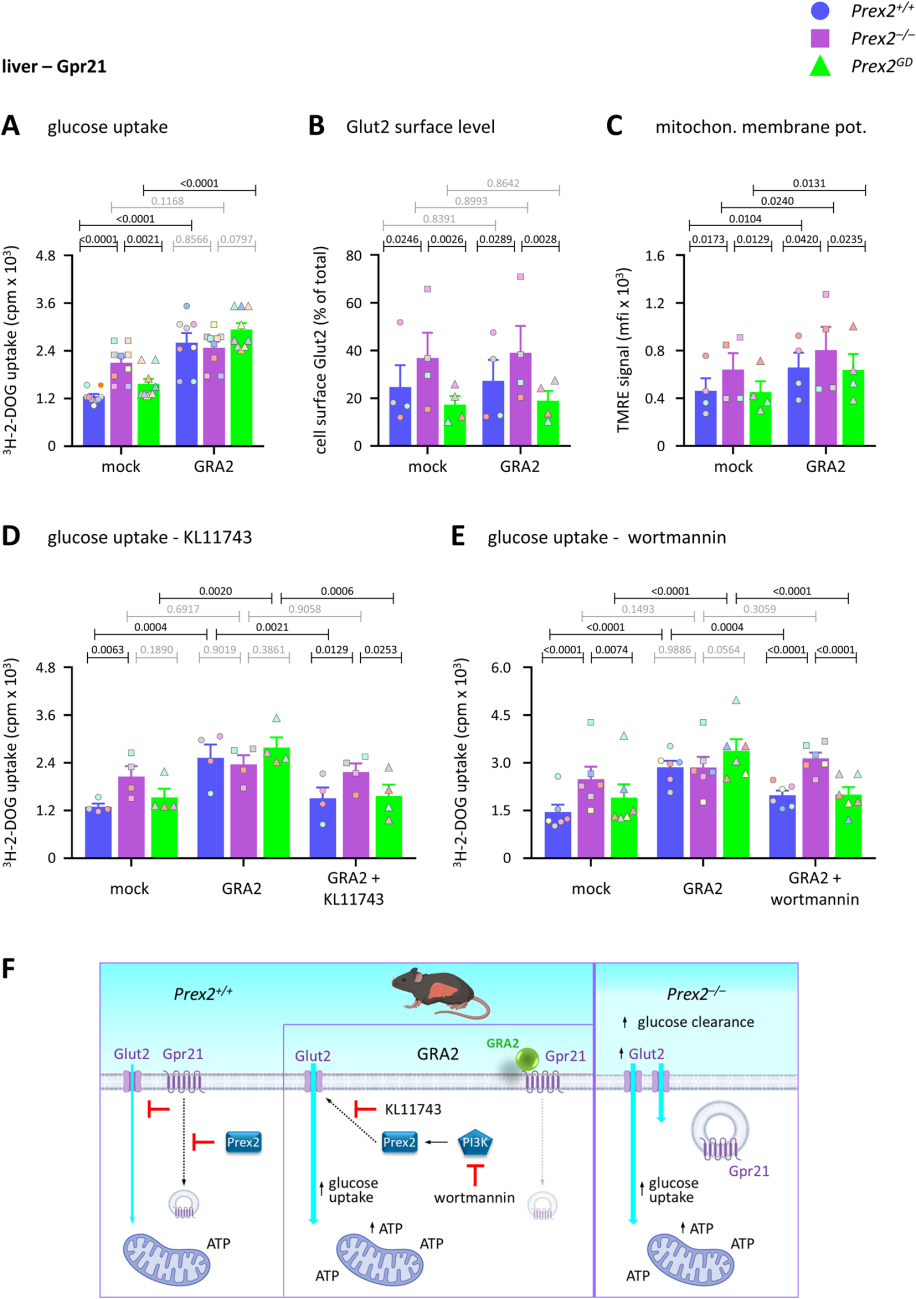
P-Rex2 limits glucose uptake into liver cells through Gpr21. (**A**) Glucose uptake. Liver cells from 15-week-old male *Prex2*^+*/*+^ (blue, circles), *Prex2*^*–/–*^ (purple, squares), and *Prex2*^*GD*^ (green, triangles) mice on chow diet were treated with 30 μM GRA2 for 3 h at 37 °C, or mock-treated, followed by the addition of 50 μM 2-DOG, 0.25 μCi ^3^H-2-DOG for 60 min. Cells were washed, lysed, and glucose uptake was measured by scintillation counting. Data are mean ± SEM of 9 independent experiments; symbol colours mark individual experiments. Statistics are two-way ANOVA with Sidak’s multiple comparisons correction. *P*-values in black denote significant differences, *p*-values in grey are non-significant. (**B**) Glut2 cell surface level. Liver cells were treated with 30 μM GRA2 for 3 h at 37 °C, or mock-treated, stained with Glut2 antibody, analysed by flow cytometry, and the mean fluorescence intensity (mfi) of Glut2 surface level expressed as % of total Glut2. Data are mean ± SEM of 4 independent experiments; symbol colours mark individual experiments. (**C**) Mitochondrial membrane potential. Liver cells were treated with 30 μM GRA2 for 30 min at 37 °C, or mock-treated, stained with MTG and TMRE, and analysed by flow cytometry for TMRE signal in MTG^+^ cells. Data are mean ± SEM of 4 independent experiments; symbol colours mark individual experiments. (**D, E**) Glucose uptake. Liver cells were pretreated with (**D**) 500 nM KL11743 or (**E**) 50 nM wortmannin for 30 min before treatment with 30 μM GRA2 for 3 h at 37 °C, or mock-treatment, followed by the addition of 50 μM 2-DOG, 0.25 μCi ^3^H-2-DOG for 60 min. Cells were washed, lysed, and glucose uptake was measured by scintillation counting. Data are mean ± SEM of (**D**) 4 and (**E**) 6 independent experiments; symbol colours mark individual experiments. Statistics (**A**–**E**) are two-way ANOVA with Sidak’s multiple comparisons correction. *P*-values in black denote significant differences, *p*-values in grey are non-significant. (**F**) Model. P-Rex2 limits hepatic glucose clearance by suppressing glucose uptake into liver cells, independently of its Rac-GEF activity, through the constitutively-active, inhibitory GPCR Gpr21. This is likely to occur through the adaptor function of P-Rex2 in regulating the trafficking of active GPCRs, which will limit the internalisation of the Gpr21, thereby enabling Gpr21 to inhibit glucose uptake. Blockade of Gpr21 activity by GRA2 removes the inhibition of glucose uptake. Use of KL11743 and wortmannin under this condition revealed P-Rex2 as a mediator of class I glucose transporter activity and PI3K activity during glucose uptake, both through adaptor functions. In *Prex2*^*–/–*^ liver cells, glucose uptake is increased because Gpr21 is internalised as the block of receptor endocytosis is removed, and Gpr21 can no longer inhibit glucose uptake.

To investigate if P-Rex2 regulates glucose uptake through Gpr21, we treated liver cells with GRA2, an inverse agonist which removes the inhibitory effect of Gpr21 on insulin signalling and glucose uptake^32,41,42^. GRA2 treatment increased glucose uptake in *Prex2*^+*/*+^ and *Prex2*^*GD*^ liver cells but did not affect the constitutively raised glucose uptake in *Prex2*^*–/–*^ cells (Fig. 5A). Therefore, P-Rex2 limits glucose uptake into liver cells through an adaptor function involving Gpr21. In contrast, GRA2 did not affect the cell surface level of Glut2 (Fig. 5B), suggesting that Gpr21 is not involved in Glut2 trafficking, and that P-Rex2 controls Glut2 trafficking independently of Gpr21. Furthermore, GRA2 treatment elevated mitochondrial membrane potential, but to a similar level in all genotypes (interaction *p*-value: 0.8163) (Fig. 5C), suggesting that Gpr21 and P-Rex2 suppress mitochondrial membrane potential independently of each other. To evaluate the role of P-Rex2 in hepatic glucose uptake further, we used KL11743, a glucose-competitive inhibitor of class 1 glucose transporters Glut1-4^43^. KL11743 had no effect on its own (Supplementary Figure S6B), but it inhibited glucose uptake in GRA2-treated *Prex2*^+*/*+^ and *Prex2*^*GD*^ liver cells, without affecting *Prex2*^*–/–*^ cells (Fig. 5D). Similarly, to assess the involvement of PI3K, we used wortmannin, an inhibitor of class 1 PI3Ks. Like KL11743, wortmannin alone had no effect (Supplementary Figure S6C), but it inhibited glucose uptake in GRA2-treated *Prex2*^+*/*+^ and *Prex2*^*GD*^ liver cells without affecting *Prex2*^*–/–*^ cells (Fig. 5E). Thus, once the suppression of hepatic glucose uptake through Gpr21 was removed by GRA2, further adaptor functions of P-Rex2 were revealed in regulating PI3K activity and class 1 glucose transporter activity upstream of glucose uptake.

Together, these data suggest that P-Rex2 suppresses glucose uptake into liver cells through the constitutively-active, inhibitory GPCR Gpr21, and independently of its Rac-GEF activity. We propose that this occurs through the recently identified adaptor function of P-Rex2 in suppressing the internalisation of active GPCRs^35^. Blockade of Gpr21 activity by GRA2 removes the inhibition of glucose uptake. In *Prex2*^*–/–*^ liver cells, glucose uptake is increased because the surface level of Glut2 is raised and presumably because Gpr21 is internalised, as it is in *Prex1*^*–/–*^ liver cells^32^, so the receptor can no longer inhibit glucose uptake (Fig. 5F).

### P-Rex2 mediates the insulin-stimulated upregulation of Glut4 and limits mitochondrial membrane potential in skeletal muscle cells

**Fig. 6:**
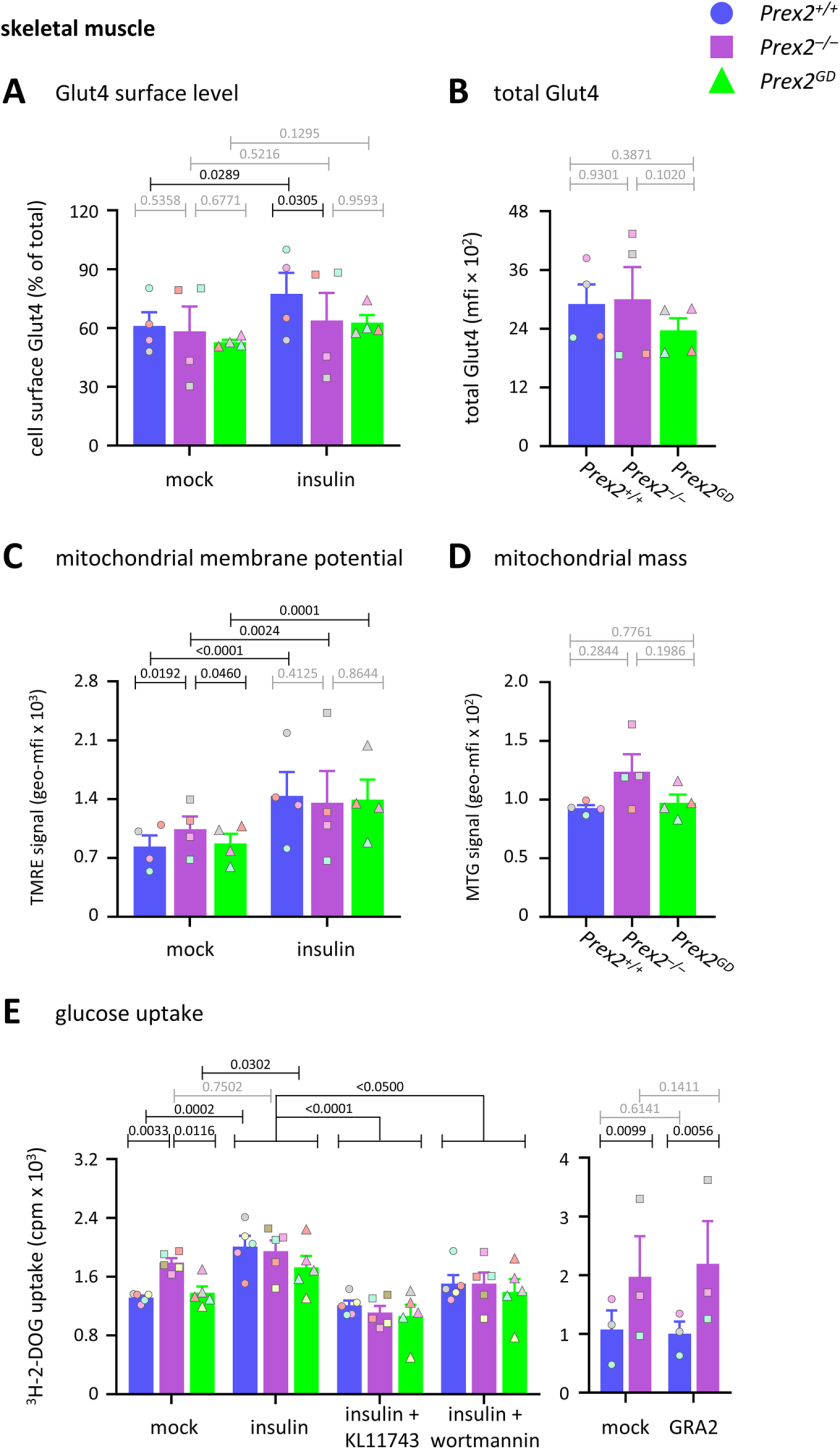
P-Rex2 mediates the insulin-stimulated upregulation of Glut4 and limits mitochondrial membrane potential in skeletal muscle cells. (**A**) Glut4 cell surface level. Skeletal muscle cells from 15-week-old *Prex2*^+*/*+^ (blue, circles), *Prex2*^*–/–*^ (purple, squares), and *Prex2*^*GD*^ (green, triangles) mice on chow diet were stimulated with 100 nM insulin for 10 min at 37 °C, or mock-stimulated, stained with Glut4 antibody, analysed by flow cytometry, and the mean fluorescence intensity (mfi) of Glut4 surface level was expressed as % of total Glut4. Data are mean ± SEM of 4 independent experiments each; symbol colours mark individual experiments. (**B**) Total Glut4 was measured as in (A) except in permeabilised cells. Data are mean ± SEM of 4 independent experiments; beige symbols show individual experiments. (**C, D**) Mitochondrial membrane potential. Skeletal muscle cells were stimulated with 100 nM insulin, or mock-stimulated, stained with MTG and TMRE, and analysed by flow cytometry for (C) TMRE signal in MTG^+^ cells and (D) MTG signal. Data are mean ± SEM of 4 independent experiments; symbol colours mark individual experiments. (**E**) Glucose uptake. Left: Skeletal muscle cells from 15-week-old male *Prex2*^+*/*+^, *Prex2*^*–/–*^, and *Prex2*^*GD*^ mice on chow diet were pretreated with 50 nM wortmannin or 500 nM KL11743 or for 30 min before stimulation with 100 nM insulin for 10 min, followed by the addition of 50 μM 2-DOG, 0.25 μCi ^3^H-2-DOG for 60 min. Cells were washed, lysed, and glucose uptake was measured by scintillation counting. Right: Skeletal muscle cells were treated with 30 μM GRA2 for 3 h, or mock-treated, followed by analysis of glucose uptake. Data are mean ± SEM of (left) 5 and (right) 3 independent experiments; symbol colours mark individual experiments. Statistics in (**A**, **C**, **E**) are two-way ANOVA with Sidak’s multiple comparisons correction. Statistics in (**B**, **D**) are one-way ANOVA with Tukey’s multiple comparisons correction. (**A**–**E**) *P*-values in black denote significant differences, *p*-values in grey are non-significant.

Next, we investigated mechanisms which might underlie the constitutively increased glucose uptake in *Prex2*^*–/–*^ skeletal muscle cells, which was overcome by insulin stimulation, as shown in Fig. 3E. The major glucose transporter in skeletal muscle cells is Glut4, which is insulin sensitive. The cell surface level of Glut4 was normal in basal skeletal muscle cells and increased upon insulin stimulation in wild type cells but not in *Prex2*^*–/–*^ or *Prex2*^*GD*^ cells (Fig. 6A). The total cellular level of Glut4 was normal in all genotypes (Fig. 6B). These data suggest that P-Rex2 mediates the insulin-stimulated upregulation of Glut4 to the surface of skeletal muscle cells through its Rac-GEF activity. However, this regulation of Glut4 cannot explain the increased glucose uptake seen in *Prex2*^*–/–*^ cells. Another glucose transporter in skeletal muscle cells is Glut1, which is insensitive to insulin. The cell surface level of Glut1 was not affected by glucose or insulin stimulation and was normal in *Prex2*^*–/–*^ and *Prex2*^*GD*^ skeletal muscle cells (Supplementary Figure S7). Hence, P-Rex2 controls glucose uptake into skeletal muscle cells but not obviously through the regulation of glucose transporter levels.

Mitochondrial membrane potential was constitutively raised slightly in *Prex2*^*–/–*^ but not in *Prex2*^*GD*^ skeletal muscle cells, despite normal mitochondrial mass in both genotypes (Fig. 6C, D). Therefore, P-Rex2 constitutively limits mitochondrial membrane potential in skeletal muscle, independently of its catalytic activity, as it does in the liver. However, unlike in hepatocytes, this was overcome by insulin stimulation (Fig. 6C).

To investigate the importance of P-Rex2 in skeletal muscle glucose uptake further, we treated skeletal muscle cells with KL11743 or wortmannin prior to insulin stimulation. As before, insulin stimulation increased glucose uptake in wild type and *Prex2*^*GD*^ cells but did not further raise the constitutively elevated response of *Prex2*^*–/–*^ cells. Both KL11743 and wortmannin inhibited insulin-stimulated glucose uptake in all genotypes, including *Prex2*^*–/–*^ cells (Fig. 6E). Furthermore, to evaluate if P-Rex2 controls glucose uptake through Gpr21, we treated wild type and *Prex2*^*–/–*^ skeletal muscle cells with GRA2. GRA2 had no effect on glucose uptake in these cells (Fig. 6E). Therefore, in skeletal muscle cells, glucose transporter and PI3K activities mediate glucose uptake independently of P-Rex2, and P-Rex2 suppresses glucose uptake independently of Gpr21, unlike in hepatocytes.

### P-Rex2 controls insulin-stimulated PIP_3_ production in liver and skeletal muscle through different mechanisms

**Fig. 7:**
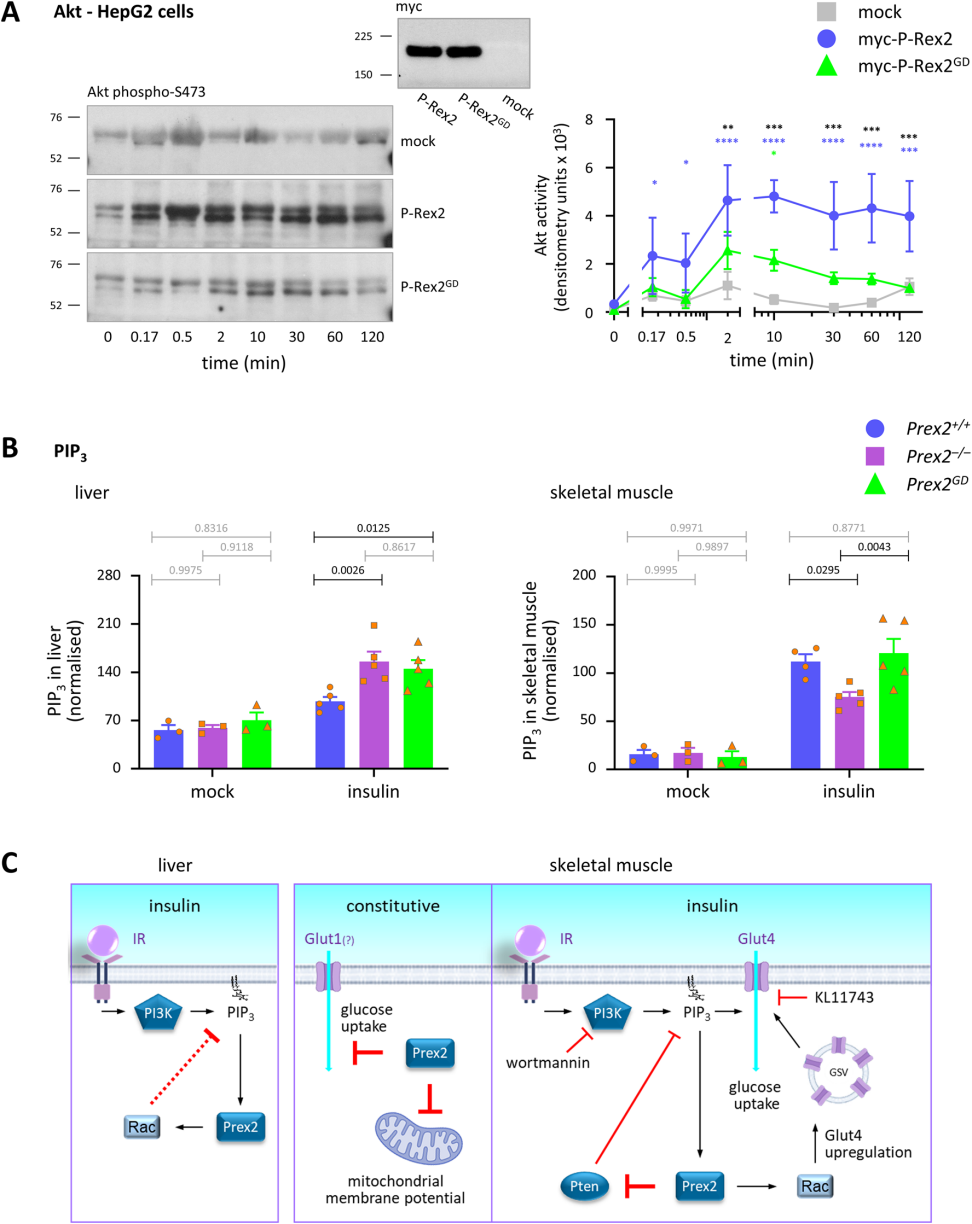
P-Rex2 controls insulin-stimulated PIP_3_ production in liver and skeletal muscle through different mechanisms. (**A**) Insulin-stimulated Akt signalling in HepG2 cells. HepG2 cells were transfected with wild type or GEF-dead pCMV3-myc-P-Rex2, or mock-transfected, as indicated, serum-starved, and stimulated with 25 nM insulin for the times shown. Left: Total cell lysates were western blotted for phospho-S473 Akt. Representative blots are shown. Middle: To control for the expression of wild type and GEF-dead P-Rex2, total lysates were probed with myc antibody. Right: Blots were quantified by Fiji densitometry. Data are mean ± SEM of 4 independent experiments. Statistics are two-way ANOVA with Sidak’s multiple comparisons correction; black stars denote significance between P-Rex2 and P-Rex2^GD^, blue stars between mock and P-Rex2, green stars between mock and P-Rex2^GD^. (**B**) PIP_3_ production: 15–19-week-old male *Prex2*^+*/*+^ (blue, circles), *Prex2*^*–/–*^ (purple, squares), and *Prex2*^*GD*^ (green, triangles) mice on HFD were fasted for 4 h and injected *i.p.* with 10 U/kg insulin or mock-treated. After 8 min, the mice were humanely killed, and liver and anterior thigh skeletal muscle were recovered. Lipids were extracted and analysed by HPLC–MS. For each cohort, PIP_3_ levels (PIP_3_/PIP_2_ ratio) were normalised to the mean *Prex2*^+*/*+^ insulin response. Data are mean ± SEM of 3 mock-stimulated and 5 insulin-stimulated mice/group pooled from two independent cohorts. Statistics are two-way ANOVA with Sidak’s multiple comparisons correction; *p*-values in black denote significant differences, *p*-values in grey are non-significant. (**C**) Model. In insulin-stimulated hepatocytes, P-Rex2 suppresses PIP_3_ production through its Rac-GEF activity, by an unknown mechanism. In skeletal muscle cells, P-Rex2 constitutively limits glucose uptake and mitochondrial membrane potential through an adaptor function. In the presence of insulin, P-Rex2 is required for the upregulation of Glut4 through its Rac-GEF activity and for PIP_3_ production through an adaptor function, likely by inhibition of Pten. Use of KL11743 and wortmannin showed that glucose transporter activity and PI3K regulate glucose uptake in skeletal muscle cells independently of P-Rex2. Gpr21 is not involved in glucose uptake by skeletal muscle cells.

As a PIP_3_-dependent Rac-GEF, P-Rex2 mediates insulin/PI3K signalling, and as an inhibitor of the tumour suppressor Pten, it regulates PIP_3_ levels^27,28,33^. We investigated if the catalytic Rac-GEF activity of P-Rex2 is required for insulin signalling and PIP_3_ production, to evaluate if its role in these pathways might be linked to its functions in glucose homeostasis. First, we overexpressed myc-tagged human wild type or GEF-dead P-Rex2 in the hepatocyte cell line HepG2, stimulated the cells with insulin, and assessed the activation of the PI3K target protein kinase B (Akt). Expression of both wild type and GEF-dead P-Rex2 increased insulin-stimulated Akt activity, despite normal total levels of Akt. However, Akt activity was significantly higher and more sustained with wild type P-Rex2 than GEF-dead P-Rex2 (Fig. 7A and Supplementary Figure S8). Hence, P-Rex2-dependent insulin/PI3K signalling was largely dependent on the Rac-GEF activity in this overexpression system. In contrast to Akt, the activity of p38 mitogen-activated protein kinase (p38 Mapk) was unaffected under the conditions tested (Supplementary Figure S8).

To assess the role of P-Rex2 in insulin/PI3K signalling and PIP_3_ production in vivo, we challenged *Prex2*^+*/*+^, *Prex2*^*–/–*^, and *Prex2*^*GD*^ mice with insulin, retrieved liver, skeletal muscle, and white adipose tissues, and tested PIP_3_ levels directly by mass spectrometry. Constitutive levels of PIP_3_ were low and equal between the genotypes in all tissues (Fig. 7B) and can therefore not explain the constitutively increased glucose uptake into *Prex2*^*–/–*^ liver and skeletal muscle cells. Insulin stimulation increased PIP_3_ production in all tissues. In the liver, insulin caused a larger increase in PIP_3_ levels in *Prex2*^*–/–*^ and *Prex2*^*GD*^ than in wild type tissue, whereas in skeletal muscle, insulin-stimulated PIP_3_ production was lower in *Prex2*^*–/–*^ compared to *Prex2*^*GD*^ and wild type (Fig. 7B). Therefore, P-Rex2 limits insulin-stimulated hepatic PIP_3_ production through its Rac-GEF activity, whereas it mediates skeletal muscle PIP_3_ production independently of its catalytic activity. In contrast, insulin-stimulated PIP_3_ production was normal in subcutaneous and visceral white adipose tissue (Supplementary Figure S9).

To summarise the role of P-Rex2 in skeletal muscle, P-Rex2 constitutively limits glucose uptake and mitochondrial membrane potential and is required for insulin-stimulated PIP_3_ production in skeletal muscle cells, independently of its catalytic activity, whereas it mediates the insulin-stimulated upregulation of Glut4 to the cell surface through its Rac-GEF activity. Its adaptor function in insulin-dependent PIP_3_ production in skeletal muscle is consistent with its role as an inhibitor of the tumour suppressor Pten (Fig. 7C).

Overall, however, there are no obvious links between the roles of P-Rex2 in PIP_3_ production and in glucose uptake, neither in the liver nor in skeletal muscle, as the former are insulin-dependent, whereas the latter are constitutive. Nor is there any link between the Rac-GEF activity of P-Rex2 and its role in glucose uptake, which it mediates through adaptor functions that involve Gpr21 in the liver but not in skeletal muscle (Figs. 5F and 7C).

## Discussion

We showed that P-Rex2 plays complex and unexpected roles in glucose homeostasis. P-Rex2 suppresses glucose clearance. It limits glucose uptake into liver cells through an adaptor function involving Gpr21 and glucose uptake into skeletal muscle cells through an adaptor function independent of Gpr21. P-Rex2 is required for insulin sensitivity and limits glucose-stimulated insulin secretion by pancreatic islets and plasma insulin levels through an adaptor function. In addition, we identified Rac-GEF activity dependent and independent roles of P-Rex2 in insulin-stimulated PIP_3_ production in liver and skeletal muscle, respectively.

The increased glucose tolerance of *Prex2*^*–/–*^ mice was seen throughout ageing and regardless of sex or diet. This role was similar to the increased glucose tolerance of *Prex1*^*–/–*^ mice which we recently described, although the latter was more variable with sex and diet^32^. The increased glucose tolerance of *Prex2*^*–/–*^ mice was unexpected, as these mice were previously reported to be glucose intolerant^27^. The difference between our findings and this previous study may in part stem from *Prex2*^*–/–*^ mouse strains having been generated independently using different targeting strategies. Also, our mice were backcrossed further to C57Bl6 genetic background, although this is unlikely to matter greatly, as glucose tolerance is similar between a range of mouse strains^44^. More important seem fasting conditions. We chose 6-h fasting prior to glucose tolerance tests to model human overnight fasting, whereas the previous study used overnight fasting, which is considered prolonged starvation in the mouse, resulting in weight loss, metabolic stress and non-physiological responses to glucose and insulin^44^.

While there were parallels, the metabolic phenotype of *Prex2*^*–/–*^ mice was overall quite different from that of *Prex1*^*–/–*^ mice. *Prex1*^*–/–*^ mice had low fasting blood glucose levels, mediated through the Rac-GEF activity^32^, whereas *Prex2*^*–/–*^ mice had normal fasting blood glucose. Furthermore, P-Rex2 and P-Rex1 mediated insulin sensitivity through different mechanisms, P-Rex1 through its catalytic activity and P-Rex2 through an adaptor function. P-Rex1 affected the duration of the insulin response, not the initial drop in blood glucose, suggesting an impaired counter-regulatory response to hypoglycaemia in *Prex1*^*–/–*^ mice rather than true insulin resistance^45,46^. In contrast, *Prex2*^*–/–*^ mice showed *bona fide* insulin resistance. This largely confirmed the previously reported insulin resistance in *Prex2*^*–/–*^ mice^27^, but again with some differences to the earlier study, which reported an impaired counter-regulatory response to hypoglycaemia rather in young *Prex2*^*–/–*^ mice on chow diet, similar to *Prex1*^*–/–*^ mice, whereas we observed true insulin resistance in old *Prex2*^*–/–*^ mice on chow diet and middle-aged mice on HFD. The difference between studies is again likely to originate at least in part from fasting conditions.

The altered glucose homeostasis in *Prex2*^*–/–*^ mice was not caused by changes in body weight, organ weight or morphology, performance in metabolic cages, or glucose excretion. It should be noted that our metabolic cages did not allow respirometry measurements, which could be done in the future. Furthermore, *Prex2*^*–/–*^ mice have normal locomotor activity, unless challenged on a rotarod device which revealed impaired motor coordination^15^. This suggested that enhanced glucose clearance underlies the increased glucose tolerance of *Prex2*^*–/–*^ mice. We identified enhanced glucose uptake in *Prex2*^*–/–*^ liver and skeletal muscle cells, but not adipocytes. This revealed another difference between the metabolic functions of P-Rex2 and P-Rex1, as P-Rex1 limits hepatic glucose uptake but has little effect on skeletal muscle^32^.

We investigated the fate of the excess glucose taken up by hepatocytes. Although histological analysis revealed a tendency towards increased glycogen storage in *Prex2*^*–/–*^ liver, this was only seen in middle-aged mice and remained within the normal range, suggesting that the excess glucose was largely metabolised rather than stored. Indeed, we saw constitutively increased mitochondrial membrane potential and ATP production in *Prex2*^*–/–*^ hepatocytes, despite normal mitochondrial mass. This is again different from *Prex1*^*–/–*^ hepatocytes, which have increased mitochondrial mass with incidences of mega-mitochondria^32^. Presumably, the liver of *Prex2*^*–/–*^ mice is less stressed than that of *Prex1*^*–/–*^ mice, because the additional increased glucose clearance into skeletal muscle relieves some of the pressure.

Our search for causes of the enhanced glucose clearance in *Prex2*^*–/–*^ mice revealed increased insulin secretion by pancreatic islets. Furthermore, although glucose had a limited effect on plasma insulin levels in wild type and *Prex2*^*GD*^ mice, likely due to the *i.p.* administration which circumvents the hepatic portal vein effect on insulin secretion^47^, it increased plasma insulin in *Prex2*^*–/–*^ mice. However, glucose uptake was raised constitutively in *Prex2*^*–/–*^ mice, independently of insulin levels, suggesting that pancreatic insulin secretion and plasma insulin are unlikely to be major contributors to the enhanced glucose clearance.

We recently discovered an adaptor role for P-Rex1 and P-Rex2 in inhibiting the agonist-stimulated internalisation of GPCRs, where the GEFs bind directly to Grk2 and control GPCR phosphorylation^35^. Furthermore, we identified the GPCR Gpr21 as a target of P-Rex1 in hepatocytes, with P-Rex1 deficiency reducing the plasma membrane localisation and increasing the endosomal localisation of the receptor and limiting glucose uptake through Gpr21^32^. Here, we showed that P-Rex2 also controls hepatic glucose uptake through Gpr21. Gpr21 is a constitutively active Gα_q_-coupled orphan GPCR important in glucose homeostasis. Early studies reporting that Gpr21 deficiency improves the glucose tolerance and insulin sensitivity of mice on HFD^48,49^ were questioned when Rapgap1 was shown to have been co-deleted accidentally^50^, but a more recent, independently generated *Gpr21*^*–/–*^ mouse with normal Rapgap1 expression also shows improved glucose tolerance^51^. We infer that P-Rex2 suppresses hepatic glucose uptake in the same way as P-Rex1, by controlling Gpr21 trafficking, enabling the receptor to inhibit glucose uptake by retaining it at the cell surface. Therefore, we propose that P-Rex2 regulates hepatic glucose homeostasis largely through Gpr21.

Presumably, the excess glucose enters *Prex2*^*–/–*^ hepatocytes through the constitutively raised cell surface level of Glut2. It remains to be seen how P-Rex2 controls Glut2 upregulation to the cell surface, as total cellular Glut2 was normal in *Prex2*^*–/–*^ hepatocytes. It seems unlikely that P-Rex2 controls Glut2 trafficking through Gpr21, as GRA2 treatment did not affect cell surface Glut2 in hepatocytes, despite increasing glucose uptake. Our experiments with KL11743 suggested that P-Rex2 suppresses hepatic glucose uptake by limiting glucose transporter activity, glucose transporter activity through an adaptor function, and use of wortmannin revealed P-Rex2 as a target of PI3K during this process.

As discussed above, P-Rex2 suppressed glucose uptake in skeletal muscle cells as well as hepatocytes. However, unlike in hepatocytes, this occurred independently of Gpr21. Furthermore, P-Rex2 constitutively limited mitochondrial membrane potential in skeletal muscle cells, but unlike in hepatocytes, this was overcome by stimulation with insulin. Moreover, we showed that P-Rex2 mediates the insulin-stimulated upregulation of Glut4 to the surface of skeletal muscle cells through its Rac-GEF activity, whereas basal Glut4 surface levels were normal. Similarly, Glut1 surface levels in *Prex2*^*–/–*^ and *Prex2*^*GD*^ skeletal muscle cells were either normal or reduced under some conditions, but never increased. Therefore, neither Glut4 nor Glut1 levels could explain the constitutively increased glucose entry into *Prex2*^*–/–*^ skeletal muscle cells. Furthermore, KL11743 and wortmannin inhibited insulin-stimulated glucose uptake in all genotypes, suggesting that P-Rex2 does not control glucose transporter activity or PI3K-dependent glucose uptake in skeletal muscle cells. Therefore, P-Rex2 limits glucose clearance in liver and skeletal muscle through distinct mechanisms.

To investigate potential underlying mechanisms in more detail, we assessed insulin signalling. Overexpression in HepG2 cells revealed that P-Rex2 promotes the insulin-stimulated activation of Akt largely through its Rac-GEF activity. However, this overexpression system did not reflect the physiologically relevant situation in vivo. In the liver, P-Rex2 limited insulin-stimulated PIP_3_ production through its Rac-GEF activity, rather than raising PIP_3_ levels. This could not explain the suppression of glucose uptake which occurred through an adaptor function of P-Rex2. However, it revealed a feedback loop from P-Rex2 to PI3K that merits further investigation in the future. Together with the wortmannin data, it suggested that PI3K has two effects on P-Rex2 in liver cells, activating its Rac-GEF activity and stimulating an adaptor function of P-Rex2 in glucose uptake when Gpr21 is inactive. In skeletal muscle, P-Rex2 was required for insulin-stimulated PIP_3_ production independently of its catalytic activity, potentially through its adaptor function in inhibiting Pten. Again, these PIP_3_ levels could not explain the increased glucose uptake in *Prex2*^*–/–*^ skeletal muscle cells.

The roles of P-Rex Rac-GEFs in glucose homeostasis differ from those known for Vav family Rac-GEFs. Whereas P-Rex1 and P-Rex2 mediate insulin sensitivity but limit glucose tolerance, the latter independently of their catalytic Rac-GEF activities, Vav2 activity is required for glucose tolerance^10^, and the metabolic phenotype of mice deficient Vav3 is diet-dependent, linked to diet-induced chronic excitation of the sympathetic nervous system^13^. Other Rac-GEFs than Prex and Vav family proteins have also been implicated in processes relevant to glucose homeostasis. For example, in vitro experiments have shown that the Rac-GEF Tiam1 is required for Glut4 translocation in the myotube-differentiated muscle cell line C2C12, and that Plekhg4 plays a similar role in adipocytes, whereas β-PIX confers glucose stimulated insulin secretion in pancreatic β cell line MIN6^2^. However, the metabolic phenotypes of mice deficient in these Rac-GEFs remain to be studied in vivo.

Overall, our study identifies complex roles of P-Rex2 in glucose homeostasis which in some ways resemble those of P-Rex1, but in most aspects are different and non-redundant. The P-Rex2 dependent suppression of glucose uptake in liver and skeletal muscle cells, and P-Rex2 dependent insulin sensitivity, are adaptor functions. P-Rex2 suppresses glucose uptake in the liver through Gpr21 and in skeletal muscle independently of Gpr21. Conceivably, P-Rex2 acts in skeletal muscle through a different inhibitory GPCR that remains to be identified.

Overall, there is little evidence supporting a major role for Pten inhibition by P-Rex2 in glucose homeostasis. To address the importance of the P-Rex2 adaptor function in inhibiting Pten specifically, as opposed to all its adaptor functions as we have done here using the *Prex2*^*GD*^ mouse, one would need to inhibit the P-Rex2/Pten interaction. However, this cannot be done in a meaningful way in a mouse model, as P-Rex2 and Pten interact through protein/protein interaction over quite large stretches involving more than one domain in each protein^16,27,28^, and introduction of the required mutations would disrupt the overall structure of P-Rex2. In contrast, the point mutations we introduced into the DH domain of P-Rex2 in the *Prex2*^*GD*^ mouse do not disrupt structure but inhibit binding to Rac^16,33,37,38,52^. In addition to the role in glucose clearance we described here, the only established adaptor function of P-Rex2 is its inhibition of Pten^28^. However, as described above, we also have recent evidence for a GEF-independent role in GPCR trafficking^35^. Furthermore, our recent study of the role P-Rex2 in melanoma showed that P-Rex2 blocked the responsiveness of tumours to therapeutics targeting the Mapk pathway through its Rac-GEF activity. Yet, in the context of activating Braf mutations and heterozygous Pten loss, which frequently occur in melanoma, *Prex2*^*–/–*^ mice but not *Prex2*^*GD*^ mice showed accelerated development of naevi, which are considered melanoma precursors^38^. Therefore, the inhibition of naevus formation is another adaptor function of P-Rex2. In light of these recent findings, it seems likely that numerous other adaptor functions of P-Rex2 exist which remain to be identified. The *Prex2*^*GD*^ mouse will be useful for elucidating such further functions.

It remains unclear whether targeting P-Rex2 would be beneficial in tackling metabolic disease, as P-Rex2 deficiency increased glucose clearance but reduced insulin sensitivity. We recently developed the first inhibitors of P-Rex Rac-GEF activity^53^, but most metabolic functions of P-Rex2 were adaptor functions, so inhibition of the Rac-GEF activity would not benefit glucose homeostasis. Targeting of P-Rex2 protein stability would conceivably increase glucose clearance but would have the undesired side-effect of compromising insulin sensitivity. In comparison, targeting the adaptor function of P-Rex2 in hepatic glucose clearance may be more promising, as one could target Gpr21 rather than the P-Rex2 protein directly, for example through therapeutics based on GRA2.

## Methods

### Mice

*Prex2*^–/–^ were previously described^15^, and were backcrossed 10 times to C57Bl6 genetic background, unless otherwise indicated. *Prex2*^–/–^ mice were compared to *Prex2*^+/+^ mice derived on the same genetic background. To minimise genetic drift, *Prex2*^–/–^ and *Prex2*^+/+^ mice were intercrossed at least once every two years. Mice were group-housed (up to five) in individually ventilated cages in the Babraham Biological Support Unit which operates as described, using 12 h light/12 h dark cycles with dawn/dusk settings^54^. For all experiments, mice were sex-and age-matched between genotypes. For in vivo experiments, they were additionally weight-matched at the onset. Prior to in vivo experiments, mice were habituated to handling by weighing, scruffing, and marking the tail with marker pen for rapid identification, for at least two days preceding the study. Mice were fed chow diet (CRM (P), Special Diets Services, Augy, France, 801722) ad libitum throughout, or were fed 45% HFD (RM AFE 45% Fat (P), Special Diets Services, 826161) ad libitum from the age of 10 weeks onwards. Unless specified otherwise, mice were assessed at the age of 15 weeks. For ageing studies, at least two cohorts of 3–5 mice per genotype, sex, and diet were followed up to one year of age. Glucose tolerance was evaluated at 10, 15, 25, 39 and 52 weeks of age, insulin sensitivity at 25 and 43 weeks, and performance in metabolic cages at 11 months. At the end of the ageing study, mice were humanely killed by CO_2_ asphyxiation and dissected for organ analysis. Breeding and experiments were conducted with approval and in accordance with the relevant guidelines from the local Animal Welfare Ethical Review Body under the British Home Office Animal Scientific Procedures Act 1986, and all relevant methods were conducted and reported in accordance with or exceeding the standards of the ARRIVE guidelines.

### Generation of Prex2^GD^ mice

We generated the catalytically inactive (GEF-dead) P-Rex2 mouse strain *Prex2*^*GD*^ as we recently described^38^. Briefly, residues Glu^22^ and Asn^204^ in the catalytic DH domain were mutated to alanine using CRISPR/Cas9 gene editing. Three sgRNAs were designed to direct wild type Cas9 to each of the relevant sites in exons 1 and 6 of the *Prex2* gene. sgRNA efficiency was assessed in vitro using the sgRNA In vitro Transcription and Screening kit (Clontech, 631439). Two sgRNAs for each exon were assessed further in NIH3T3 cells (ATCC, CRL-1658; see ‘cell culture’ section) for their efficacy using the Surveyor Mutation Detection Kit (Integrated DNA Technologies, 706025). sgRNAs GCGCGACTACGTGGGCACGC-*TGG* and GTAATGAGGCCAAGAGACAGA-*TGG* (adjacent PAM sequences in italics) were chosen for targeting exon 1 and exon 6, respectively. 200 bp ssDNA repair templates were designed to introduce the desired point mutation by homology-directed repair and were purchased from Dharmacon as PAGE-purified Ultramer ssDNAs. The selected sgRNA, ssDNA repair template and Cas9 mRNA were microinjected into the pronucleus of C57BL/6J mouse zygotes by the Babraham Gene Targeting Facility. Initially, mice carrying the E22A mutation were generated. Homozygous *Prex2*^*E22A/E22A*^ animals were then subjected to a further round of pronuclear injections to add the *Prex2*^*N204A*^ mutation. Sequences which confirm the targeting of the *Prex2*^*GD*^ mouse strain are accessible at GenBank, accession numbers PQ488550 for E22A and PQ488549 for N204A, and traces can be viewed at Zenodo, doi.org/10.5281/zenodo.14001235. Homozygous double knock-in *Prex2*^*GD*^ (*Prex2*^*E22A/E22A;N204A/N204A*^) mice bred well and were born at the expected Mendelian rate, fertile, and apparently healthy. Once the *Prex2*^*GD*^ strain was fully established, genotyping was done by Transnetyx (Cordova, TN, United States). *Prex2*^*GD*^ mice were compared to *Prex2*^+/+^ and *Prex2*^–/–^ mice on the same C57BL/6 J genetic background.

### Cell culture

All mammalian cell lines used were purchased from ATCC (www.atcc.org) and were maintained at 37 °C, 5% CO_2_, in a humidified atmosphere and used between passages 4 and 25. Aliquots of early-passage cells were stored in liquid N_2_. Human hepatocellular carcinoma HepG2 cells (ATCC, HB-8065) were cultured in flasks (Nunc) in Dulbecco’s Modified Eagle’s Medium (DMEM) (Invitrogen, 41965–039) supplemented with 10% heat-inactivated foetal bovine serum (FBS) (Invitrogen, 10270–106), unless stated otherwise, and were subcultured twice weekly using 0.25% (w/v) Trypsin, 0.53 mM EDTA. NIH/3T3 mouse fibroblasts (ATCC, CRL-1658) were cultured in flasks in ATCC-formulated DMEM (ATCC, 30-2002) with bovine calf serum (ATCC, 30-2030) and subcultured twice weekly using Trypsin/EDTA. For transient overexpression, transfection was done using JetPEI (Polyplus, 101-10N) following the manufacturer’s protocol.

### Glucose tolerance

Mice were fasted for 6 h by moving into a fresh cage without food at 8.30 am, water remaining available ad libitum. 15 min prior to glucose injection, the mice were weighed and their baseline fasting blood glucose level was assessed using an AlphaTRAK 2 glucometer (Abbott). At 2.30 pm, 2 g/kg glucose (20% glucose, Gibco, A2494001; 10 ml/kg) was administered by intraperitoneal (*i.p.*) injection. Tail bleeds were performed at 15, 30, 45, 60, 75 and 90 min after glucose injection and the blood glucose level determined by glucometer. For each timepoint, two glucometer readings were taken, and the lowest value recorded. Tail bleeds were done by pricking the tip of the tail (rather than the lateral tail vein) with a sterile needle for the first bleed, dislodging the scab for each following bleed, and without restraining the animal, to minimise stress and discomfort. Each bleed was no more than a few drops in blood volume. Mice on HFD at 39 and 52 weeks of age were injected with a lower dose of glucose (1 g/kg; 20% glucose at 5 ml/kg), to prevent dangerous levels of sustained hyperglycaemia.

### Insulin sensitivity

Mice were fasted for 4 h by moving into a fresh cage without food at 8.30 am, water remaining available ad libitum. 15 min prior to insulin injection, the mice were weighed, and their fasting blood glucose levels tested by glucometer as described here-above. At 12.30 pm, 0.75 IU/kg sterile human recombinant insulin (Actrapid, Novo Nordisk, A10AB01) in DPBS (5 ml/kg at 0.15 IU/ml) was administered by subcutaneous (*s.c.*) injection. Tail bleeds were performed at 15, 30, 45, 60, 90, 120 and 180 min after insulin injection and the blood glucose level determined by glucometer. For *Prex1*^*GD*^ cohorts and their controls, a reduced insulin dose of 0.375 IU/kg was used. In rare cases, animals reached dangerously low blood glucose levels of 2–3 mM after insulin challenge. These mice were immediately injected *i.p.* with 100–150 μl 20% glucose to avoid hypoglycaemic shock, monitored, and excluded from the rest of the experiment.

### Metabolic cages

To monitor food and water consumption and the production of urine and faeces, mice were group-housed (up to 3) in metabolic cages (Techniplast, UK), at the age of 11 months. Mice were habituated to the metabolic cages for 1 h on the first and 3 h on the second day before the experiment, then housed in the metabolic cages for 16 h overnight on 3 subsequent nights with pre-weighed food and water ad libitum. During daytime, the mice were returned to their home cages. The weights of urine and faeces and of the remaining food and water were recorded after each night. The metabolic cages were cleaned and stocked with fresh pre-weighed food and water before each night.

### Organ weights and histology

Mice of the indicated ages were humanely killed by CO_2_ asphyxiation (death confirmed by severing the femoral artery), weighed, and dissected for collection of brain, interscapular BAT, inguinal WAT, anterior thigh skeletal muscle, pancreas, kidneys, spleen, liver, thymus, heart, lung, salivary gland, and where applicable testes. The liver was scored visually for steatosis, from grade 0, with a healthy dark red colour, to grade 3, with a severe level of fat and yellow in colour overall. Organs were weighed and where required snap-frozen in liquid N_2_. For histological analysis, freshly dissected liver, skeletal muscle, inguinal WAT and interscapular BAT were fixed in formalin, and H&E sections were prepared by Abbey Veterinary Services and analysed by pathologist Dr Cheryl Scudamore (ExePathology, Exmouth, UK) by blinded review using a grading system from 0 to 5, where 0 is normal and 5 the whole tissue affected.

### Tissue and cell lysates, and western blotting

For preparation of cerebellar lysates, cerebellum was dissected and immediately snap-frozen in liquid N_2_. The frozen tissue was wrapped in cling film, shattered using a hammer, weighed, and lysed in 9 vol RIPA buffer (30 mM Hepes, pH 7.4, 150 mM NaCl, 1% Nonidet P-40, 0.5% deoxycholate, 0.1% SDS, 5 mM EGTA, 4 mM EDTA) supplemented with 1 mM DTT, 100 μM PMSF, and 10 μg/ml each of leupeptin, pepstatin-A, aprotinin and antipain (RIPA^+^). Samples were incubated for 5 min on ice, or triturated frequently and incubated for 10 min on ice, then centrifuged at 800 × g for 5 min at 4˚C. Boiling 4 × SDS-PAGE sample buffer was added to the supernatant (to final 1.3 ×), and samples were boiled for 5 min, snap frozen liquid N_2_, and stored at -80˚C. To prepare lysates of cultured cells, cells were rinsed in ice-cold phosphate buffered saline (PBS, Invitrogen, 70011–036) and scraped into RIPA^+^ buffer. Samples were transferred into pre-cooled Eppendorf tubes, incubated on ice for 5 min, and debris was sedimented at 12,000×*g* for 10 min at 4 °C. Boiling 4 × SDS-PAGE sample buffer was added to the supernatant to final 1.3×, and samples were boiled for 5 min, snap frozen, and stored at -80˚C.

For western blotting, proteins were resolved by SDS-PAGE and blotted onto Immobilon-P membrane (Millipore, IPVH00010). Primary antibodies were P-Rex2 (affinity-purified rabbit polyclonal ‘78’, 1:30,000)^15^, myc (clone 9E10, Babraham Bioscience Technologies, 1:50), phospho-Akt S473 (Cell Signaling Technology, 9271, 1:500), total Akt (Cell Signalling Technology, 9272, 1:1000), and phospho-p38 Mapk T180/Y182 (Cell Signaling Technology 9211, 1:500). Secondary antibodies were goat anti-mouse IgG-Horseradish Peroxidase (HRP) conjugate (BioRad, 1:3000) or goat anti-rabbit IgG-HRP (BioRad, 1:3000). Amersham ECL or ECLPlus were used for detection. To control for protein loading, blots were stained with 0.1% Coomassie Brilliant Blue R-250. Where required, blots were stripped in 25 mM glycine, pH 2.0, 1% SDS and reprobed. Western blots were quantified by densitometry using Fiji (ImageJ).

### ELISA

To analyse plasma insulin, mice were fasted from 8.30 am for 6 h, challenged with glucose as described for the glucose tolerance tests, or mock-treated, and killed humanely by CO_2_ asphyxiation 15, 60 or 90 min later. Peripheral blood was collected immediately by cardiac puncture into 300 µl EDTA K2E microvettes (Sarstedt, 16.444.100). Samples were centrifuged for 5 min at 2000 × g at RT, and the supernatant was transferred into fresh tubes and centrifuged again. The second supernatant (plasma) was aliquoted and stored at -80˚C. To quantify plasma insulin, the Ultra-Sensitive Mouse Insulin (Crystal Chem, 90080) ELISA kit was used following the manufacturers’ guidelines. Insulin secreted from pancreatic islets (see below) was measured using the same kit. To analyse glucose in urine, mice were fasted and challenged with glucose as described for the glucose tolerance test, scruffed 90 min after glucose injection, and urine was collected into an Eppendorf tube. A mouse glucose ELISA (CrystalChem, 81692) was used according to the supplier’s protocol.

### Insulin release from isolated pancreatic islets

Pancreatic islet isolation was adapted from^55^. 15-week-old mice were humanely killed by cervical dislocation and death confirmed by pithing. The pancreas was recovered, injected with 5 ml ice-cold collagenase XI (1000 U/ml, Sigma, C7657) in HBSS (Gibco, 14185–052) with 1 mM CaCl_2_, and placed into a 50 ml falcon tube which was swirled in a 37 °C water bath for 20 min, with shaking every 3 min for 1 min. Ice-cold HBSS with 1 mM CaCl_2_ was added to give a volume of 30 ml, and samples were centrifuged at 326×*g* for 2 min. Samples were washed again, resuspended in 5 ml HBSS with 1 mM CaCl_2_, slowly added on top of 7.5 ml Histopaque 1077 (Sigma, 10771), centrifuged at 2000×*g* for 15 min, brake 0, and the middle layer containing islets was transferred into a 5 cm dish.

20 islets were hand-picked under a dissection microscope using a sterile wide-neck 1 ml plastic pipette, placed into each well of a 6-well plate containing Dutch-modified RPMI 1640 (Gibco, 22409015) with 10% FBS, 1% L-glutamine, 100 U/ml penicillin and 100 μg/ml streptomycin**,** and left to recover ON in a humidified 37 °C, 5% CO_2_ incubator. 5 or 10 islets, as stated, were handpicked into 2 ml Eppendorf tubes containing 1 ml RPMI 1640 without glucose (Gibco, 11879020), the volume adjusted to 2 ml, and samples were left to recover for 1 h in a humidified 37 °C, 5% CO_2_ incubator, before being placed into a thermomixer at 37 °C without shaking. A 50 µl aliquot was removed, taking care not to dislodge the islets resting at the bottom, and snap-frozen in liquid N_2_. 50 µl RPMI 1640 containing 80 mM glucose was added (for 2 mM final), and tubes were gently inverted twice to ensure mixing and were incubated for 15 min. After 14 min, the tubes were inverted again, and at 15 min, once the islets had sunk to the bottom, another aliquot was taken. 50 µl fresh media containing 720 mM glucose (for 20 mM final) was added for a further 45 min, after which a final aliquot was taken. Secreted insulin was quantified by ELISA as described here-above.

To determine the total amount of insulin in pancreatic islets, 5 islets were sedimented for 5 min at 326 × g at 4 °C and lysed in 1 ml ice-cold RIPA^+^ buffer by incubation on ice for 5 min with frequent vortexing. Samples were centrifuged at 10,000 × g for 3 min at 4 °C, and the supernatant was snap-frozen in liquid N_2_. These samples were diluted 1:10 before analysis by insulin ELISA.

### Isolation of primary liver cells, adipocytes, and skeletal muscle cells

To isolate liver cells, mice were culled by cervical dislocation, death was confirmed by pithing, and the liver perfused with PBS by cannulating the hepatic portal vein using a 25G needle, followed by cutting the inferior vena cava to drain blood and perfusion fluid. The perfused liver was quickly excised, minced using scissors, rinsed in DMEM for 30 min at RT with gentle agitation, passed through a 70 μm cell strainer (Fisher Scientific, 11597522), and rinsed with 5 ml DMEM. Cells were sedimented at 100 × g for 3 min at RT, resuspended in Krebs Ringer Buffer (KRB; 12 mM Hepes, pH 7.4, 121 mM NaCl, 4.9 mM KCl, 1.2 mM MgSO_4_, 5 mM CaCl_2_) containing 0.1% BSA and incubated at 37 °C for 90 min prior to use. Liver cells were counted by haemocytometer and viability assessed by Trypan Blue (Sigma Aldrich, T8154) exclusion. For Seahorse assays, liver cells were resuspended in DMEM, 10% FBS, 100 U/ml penicillin and 100 μg/ml streptomycin.

The isolation of mature adipocytes was adapted from^56^. Mice were humanly killed by CO_2_ asphyxiation, death was confirmed by severing the femoral artery, and mouse adipose tissues (BAT, visceral WAT and subcutaneous WAT) were carefully excised, minced using scissors, and digested in a 50 ml Falcon tube containing 10–15 ml digestion buffer (KRB with 3% BSA, 1 mg/ml type II collagenase (Gibco, 17101–015), 4 mM glucose, and 500 nM adenosine) for 1 h at 37 °C, with gentle inverting every 10 min. EDTA was added to final 2 mM, and the homogenate was passed through a 200 μm cell strainer (Cambridge Bioscience, 43-50200-50). To collect mature adipocytes specifically, mature adipocytes were allowed to float to the top of the filtered homogenate for 1–2 h at RT, and the floating white cell layer was carefully harvested using a plastic Pasteur pipette. Adipocytes were counted using a haemocytometer, resuspended in KRB, 0.1% BSA, and incubated in a 12-well plate for 3 h at 37 °C, 5% CO_2_ prior to use.

To isolate skeletal muscle cells, 1.5 g medial gastrocnemius, semimembranosus, and rectus femoris muscles were collected from mouse hind limbs, minced into 2–4 mm pieces using scissors, and dissociated by incubation with Skeletal Muscle Dissociation Kit enzymes (Miltenyi Biotec, 130-098-305) according to the manufacturer’s instructions for 30 min at 37 °C in a GentleMACS C Tube under continuous gentle rotation, followed by homogenisation in a GentleMACS Dissociator (Miltenyi). The skeletal muscle cells were filtered through a 70 μm cell strainer, sedimented at 300 × g for 20 min, resuspended in KRB, 0.1% BSA, and incubated for 90 min at 37 °C prior to stimulation. Skeletal muscle cell viability was typically 80%. Mature muscle fibres were largely dissociated, but myoblasts and myotubes in elongated bundles remained intact, the latter making up ~ 60–70% of total cell number recovered.

### Glucose uptake

Glucose uptake assays were adapted from^29^ and were performed with freshly isolated liver cells, skeletal muscle cells and mature adipocytes. For liver, 1 × 10^6^ liver cells in 200 µl KRB, 0.1% BSA in Eppendorf tubes were stimulated with 200 µl 2 × insulin (Actrapid, 100 nM final) for 10 min at 37 °C, or mock-stimulated with KRB, 0.1% BSA, followed by the addition of 200 µl of 3 × 2-deoxyglucose (2-DOG; Cayman, 14325, for 50 μM final) and 0.25 μCi ^3^H-labelled 2-DOG (PerkinElmer, NET328250UC) and further incubation for 60 min. Alternatively, liver cells were treated with 30 μM GRA2, for 3 h at 37 °C, or mock-treated with KRB, 0.1% BSA, before further treatment as here-above. In some experiments, liver cells were pretreated with 500 nM KL11743 or 50 nM wortmannin for 30 min before the GRA2 treatment. Liver cells were washed three times in ice-cold KRB and lysed in RIPA buffer for 5 min on ice. Lysates were mixed with Ultima Gold scintillation fluid (Perkin Elmer, 6013326), and glucose uptake was measured by scintillation counting (Packard 1600TR, Liquid Scintillation Analyser).

Mature adipocytes (isolated from visceral, subcutaneous white, or brown adipose tissue) in a 12-well plate at 1 × 10^6^ cells per well in 1 ml KRB, 0.1% BSA were stimulated with 250 µl 5 × insulin (for 100 nM final) for 10 min at 37 °C, or were mock-stimulated with KRB, 0.1% BSA, followed by the addition of 250 µl 6 × 2-DOG (for 50 μM final) and 0.25 μCi ^3^H-2-DOG, and further incubation for 60 min. 1 ml liquid was carefully aspirated from the bottom of the well, and 1.5 ml ice-cold KRB added to the floating cells. 1.5 ml of liquid was again removed and replaced with fresh buffer, and this wash was repeated 5 times. After the final aspiration, 250 µl RIPA buffer was added, and samples were incubated on ice for 5 min and vortexed hard. Glucose uptake was measured as described for liver cells.

Skeletal muscle cells from 60 mg of tissue in 200 μl KRB, 0.1% BSA were stimulated with 100 nM insulin for 10 min at 37 °C in Eppendorf tubes, or mock-stimulated with KRB, 0.1% BSA, followed by the addition of 50 μM 2-DOG and 0.25 μCi ^3^H-2-DOG, and further incubation for 60 min. In some experiments, skeletal muscle cells were pretreated with 500 nM KL11743 or 50 nM wortmannin for 30 min before the insulin stimulation. Cells were washed three times in ice-cold KRB by centrifugation at 1500×*g* for 3 min at 4 °C and lysed in RIPA buffer for 5 min on ice. Glucose uptake was measured as measured as described here-above.

### Cell surface level of glucose transporters

The cell surface levels of glucose transporters were evaluated in liver and skeletal muscle cells using flow cytometry. 1 × 10^6^ liver cells in 200 µl KRB, 0.1% BSA or skeletal muscle cells from 60 mg of tissue in 200 µl KRB, 0.1% BSA were stimulated with 5 mM glucose or 100 nM insulin for 10 min at 37 °C, or were mock-stimulated with KRB, 0.1% BSA, and sedimented at 300 × g for 5 min at 4 °C. Alternatively, cells were stimulated with 100 nM insulin for 10 min at 37 °C cells, and 5 mM glucose was added for another 30 min, or cells were treated with 30 μM GRA2 for 3 h at 37 °C, or mock-treated with KRB, 0.1% BSA, and sedimented. Cells were stained with antibodies to Glut2 (Santa Cruz, sc-518022-AF647, 1:200), Glut4 (Novus Biologicals, NBP1-49533F, 1:200), or Glut1 (Fisher Scientific, PA146152, 1:200), as appropriate, in the presence of Fc block (BD Biosciences, 553,142, 1:1000) for 30 min on ice in the dark, washed three times with ice-cold PBS, and resuspended in 200 μl PBS. Flow cytometry was performed using a BioRad ZE5 flow cytometer, recording 20,000 events per sample. The mean fluorescence intensity (mfi) of Glut2, Glut4, and Glut1 signals were quantified using FlowJo. To determine total cellular levels of the glucose transporters, liver cells were permeabilised in 0.1% Triton X-100/PBS for 10 min on ice, and skeletal muscle cells were permeabilised in 0.2% Triton 100/PBS for 20 min on ice, prior to washing twice in PBS and staining for flow cytometry.

### ATP production (Seahorse)

ATP production was analysed by measuring oxygen consumption rate (OCR) and extracellular acidification rate (ECAR) in an Agilent Seahorse XF96 analyser by real-time ATP rate assay (103,592–100), according to the manufacturer’s protocol. Unless otherwise stated, all reagents were from Agilent Technologies. 200 μl liver cells in DMEM, 10% FBS, 100 U/ml penicillin and 100 μg/ml streptomycin were plated at 2 × 10^4^/well in a poly-D-lysine (0.1 mg/ml)-coated 96-well plate (101,085–004) and incubated ON in a humidified incubator at 37 °C, 5% CO_2._ A sensor cartridge was hydrated overnight at 37 °C. Cells were rinsed and incubated in prewarmed assay medium (DMEM [103575–100, pH 7.4], 10 mM glucose [103577–100], 1 mM pyruvate [103578–100], 2 mM glutamine [103579–100]) at 37 °C for 45–60 min. 1.5 μM oligomycin and 0.5 μM rotenone/antimycin A were loaded into the sensor cartridge according to the manufacturer’s instructions for induced ATP-rate assay. The Seahorse analyser was run using the standard initial equilibration step, then incubation for 2 h with readings taken every 10 min, followed by the addition of oligomycin for 18 min and then rotenone for another 18 min, with readings taken every 6 min. ATP production from glycolysis and mitochondrial respiration was analysed using WAVE software (Agilent Technologies).

### Mitochondrial membrane potential

To evaluate mitochondrial membrane potential by flow cytometry, 1 × 10^6^ liver cells or skeletal muscle cells from 60 mg tissue in 200 µl KRB, 0.1% BSA were stimulated with 5 mM glucose or 100 nM insulin or were treated with 30 μM GRA2 for 30 min at 37 °C, or mock-treated with KRB, 0.1% BSA, before ice-cold PBS was added. Liver cells were sedimented at 300 × g for 5 min at 4 °C, and skeletal muscle cells were sedimented at 1500 × g for 3 min at 4 °C. Cells were stained with cell-permeable MitoTracker Green (MTG, Invitrogen, M7514, 100 nM) and tetramethyl-rhodamine ethyl ester (TMRE, Abcam, ab113852, 200 nM) dyes for 30 min on ice in dark and washed three times with ice-cold PBS. Flow cytometry was performed using a BioRad ZE5 flow cytometer, recording 20,000 events per sample. The mfi of MTG and TMRE were quantified using FlowJo.

To evaluate mitochondrial membrane potential by immunofluorescence microscopy, 200 µl liver cells in DMEM were plated into poly-D-lysine (0.1 mg/ml)-coated ibidi µ-slides (ibidi, 80826) at 2 × 10^6^/well for 2 h in a humidified incubator at 37 °C, 5% CO_2_. The medium was removed, MTG, TMRE, and Hoechst 33342 (Thermo Fisher Scientific, 62294, 1:400) dyes were added, and the cells were live-imaged using a Nikon Eclipse Ti-E widefield system.

### Insulin signalling pathway analysis

To evaluate insulin signalling in HepG2 cells, cells were transfected with wild type or GEF-dead pCMV3-myc-P-Rex2^18,37^, or mock-transfected with empty pCMC3-myc vector^57^, serum-starved for 8 h in serum-free medium containing 0.1% fatty-acid-free BSA, and stimulated with 25 nM insulin for various periods of time from 0 to 2 h. Cells were sedimented at 10,000 × g for 30 s at 4 °C and lysed in RIPA^+^ buffer for 5 min on ice with frequent vortexing. Debris was sedimented at 12,000 × g for 3 min at 4 °C. Boiling 4 × SDS-PAGE sample buffer was added to the supernatant (to final 1.3 ×), and samples were boiled for 5 min, snap frozen liquid N_2_, and stored at -80˚C. Total cell lysates were western blotted for active Akt (phospho-S473), total Akt, and active p38 Mapk (phospho-T180/Y182). To control for the expression of wild type or GEF-dead P-Rex2, blots were probed with myc antibody.

### PIP_3_ production

Mice were fasted as described for the insulin sensitivity test, and injected *i.p.* with 10 U/kg insulin (Actrapid, 5 ml/kg at 2 IU/ml in DPBS) or mock-treated with DPBS. After 8 min, mice were humanely killed by cervical dislocation, death confirmed by pithing, and dissected rapidly to recover subcutaneous adipose tissue, anterior thigh skeletal muscle, visceral fat, and liver. The liver and skeletal muscle were washed in DPBS. All organs were snap-frozen in liquid nitrogen and stored at -80˚C. Organs were ground under liquid N_2_ using a pestle and mortar, and 2–5 mg of liver and skeletal muscle tissues and 7–11 mg of the white adipose tissues were weighed into 2 ml Eppendorf tubes.

To extract lipids from adipose tissues, 500 μl of samples at 10 mg/ml were added to 225 μl CHCl_3_:MeOH (1:2 v:v) followed by 195 μl H_2_O. Samples were vortexed, and 10 ng of deuterated stearoyl-arachidonoyl-PIP_3_ was added as an internal standard, to control for any loss of lipids during the extraction process. 725 µl CHCl_3_ was added, samples were centrifuged at 2348 × g for 5 min, and the bottom organic layer was discarded. This process was repeated once. 500 μl CHCl_3_:MeOH (1:2) was added, followed by 170 μl 2 M HCl, then 500 μl CHCl_3_, and the samples were vortexed and centrifuged again. The bottom layer was added to 708 μl of the upper-aqueous phase of pre-derivatisation wash (CHCl_3_:MeOH:0.01 M HCl, 2:1:0.75 v:v:v). The samples were vortexed, centrifuged again, and the bottom layer was added to 100 μl CHCl_3_:MeOH (1:2). To extract lipids from liver and skeletal muscle, tissues were resuspended at 5 mg/ml in ‘kill mix’ (MeOH:CHCl_3_:1 M HCl 2:1:10.276 v:v:v) containing H_2_O at a ratio of 750:170. 100 μl of the samples were further diluted, by adding to 820 μl kill mix:H_2_O, before 10 ng PIP_3_ and PI were added as internal standards. 725 μl CHCl_3_ and 170 μl 2 M HCl were added, and samples were vortexed and centrifuged as above. The bottom organic layer was added to 700 μl upper aqueous phase of pre-derivatisation wash. Samples were vortexed, centrifuged again, and the bottom layer was recovered.

Lipid phospho-groups were methylated to protect them during the HPLC–MS. In a chemical fume cupboard, 50 µl 2 M trimethylsilyl (TMS)-diazomethane in hexanes (Sigma Aldrich, 362,832) was added for 10 min, ensuring there was adequate quench solution (MeOH:CH3COOH, 3:1 v:v) for utensils which came into contact with TMS-diazomethane. 6 µl glacial acetic acid was added and samples were washed twice with the upper aqueous phase of CHCl_3_:MeOH:H_2_O (2:1:0.75 v:v:v). 100 µl 90% MeOH was added, and samples were dried in mass spectrometry vials under N_2_, re-dissolved in 100 µl 80% MeOH, sonicated, and stored at -80 °C. The extracted lipids were analysed by high performance liquid chromatography-mass spectrometry (HPLC–MS) as described^58^.

### Data collection and statistical analysis

Sample size was determined using power calculations to yield 80% power, based on results of pilot experiments and on previously published data as referenced. Experiments were performed at least three times except where indicated. Sample size and numbers of independent experiments are detailed in figure legends. Animals for experimental cohorts were selected as described under ‘Mice’ according to genotype, group size, sex, age, and weight. Within these criteria, mice were selected for cohorts at random by the staff of the Biological Support Unit. Image analysis was performed in a blinded manner. Statistical analysis and plotting of graphs were performed in GraphPad Prism 10. Data were evaluated for normality of distribution to determine if parametric or non-parametric methods of analysis were appropriate. Where warranted, data were log-transformed or square-root transformed prior to statistical analysis. For comparison of two groups, unpaired or paired Student’s t-test was used, as appropriate, whereas for comparison of multiple groups, one-way or two-way ANOVA was used, as appropriate, followed by post-hoc test with multiple comparisons correction. Data in categories were analysed by chi-square test. Where data were normalised, statistical analysis was done on raw data, prior to normalisation. Parameters with values of *p* ≤ 0.05 were considered to differ significantly. In the figures, results are presented as mean ± standard error of the mean (SEM). *P*-values in black denote significant differences, *p*-values in grey are non-significant. In time courses, * indicates *p* < 0.05, **p < 0.01, ***p < 0.001, and ****p < 0.0001. Where groups of individual mice were compared, symbols in graphs are coloured beige; where data are expressed as mean of individual experiments with matching/pairing by genotype/condition/experiment, symbols are colour-coded by experiment. The number of experimental repeats is indicated in the figure legends.

## Supplementary Information

Supplementary Information.

## Data Availability

The data that support the findings of this study are available from the corresponding author upon reasonable request. The sequences which confirm the targeting of the Prex2GD mouse strain are accessible at GenBank, accession numbers PQ488550 for mutation E22A and PQ488549 for mutation N204A, and the sequencing traces can also be viewed at Zenodo, doi.org/10.5281/zenodo.14001235.
