# Supplementary material for: P-Rex2 suppresses glucose uptake into liver and skeletal muscle through different adaptor functions
Source: Sci Rep. 2025 Aug 5;15:25770. doi: 10.1038/s41598-025-01720-w (PMC12325715; doi:10.1038/s41598-025-01720-w)

Supplementary Information

P-Rex2 suppresses glucose uptake into liver and skeletal muscle through different adaptor functions

Elpida Tsonou [1, 2, 3], Julia Y. Chu [1, 2], Polly A. Machin [1, 2], Anna G. Roberts [2], Anne Segonds-Pichon [4], David Baker [3], David C. Hornigold [3] and Heidi C. E. Welch [2]

[1] These authors contributed equally to the project and share first authorship.

[2] Signalling Programme and [4] Bioinformatics Facility, The Babraham Institute, Cambridge, UK

[3] Bioscience Metabolism, Research and Early Development, Cardiovascular, Renal and Metabolism (CVRM), BioPharmaceuticals R&D, AstraZeneca, Cambridge, UK

Content:

Supplementary Figures 1-9

Uncropped blots

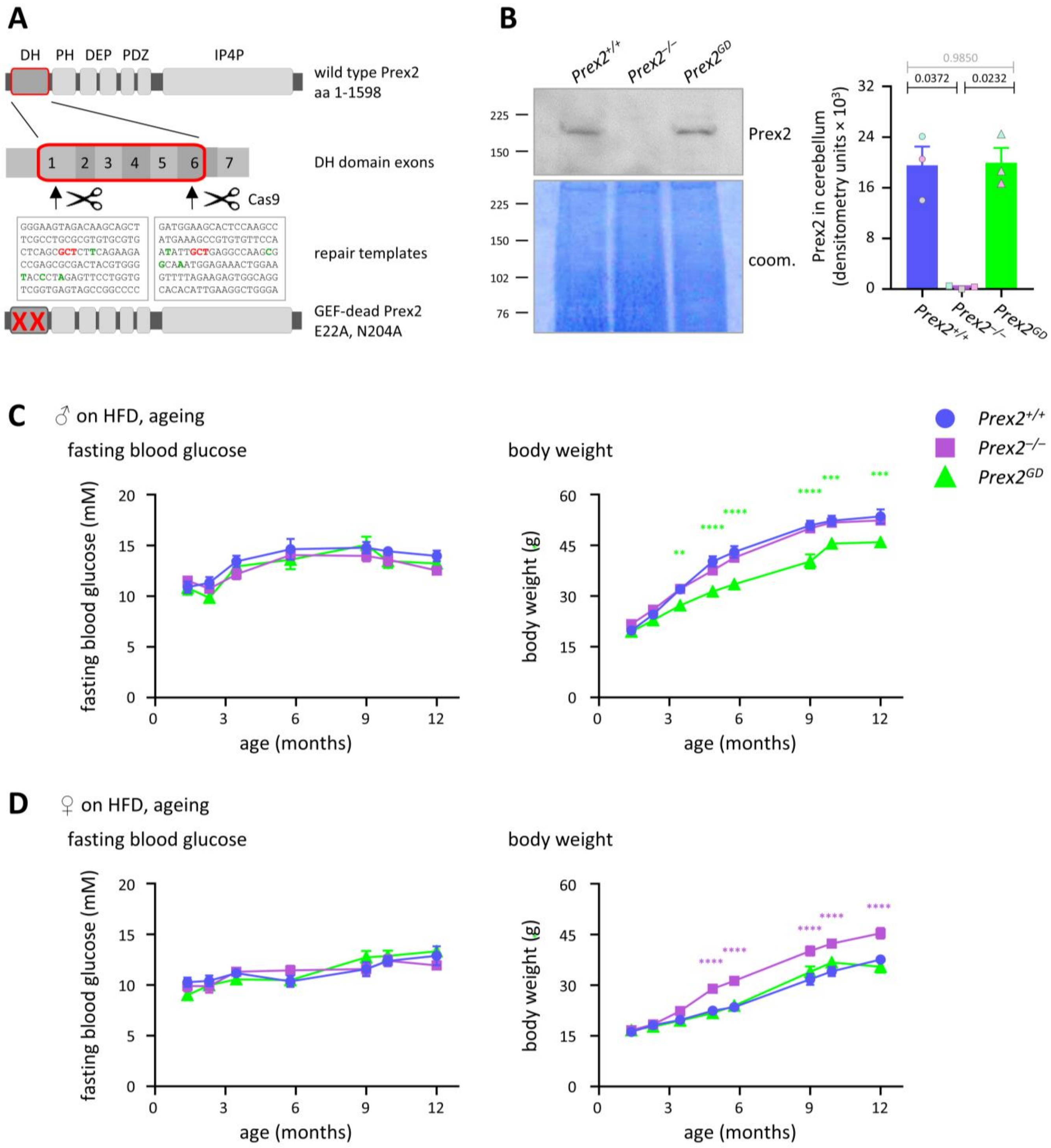

Supplementary Figure 1. Generation of *Prex2*^{GD} mice. (A) Schematic of the targeting strategy. Cas9 nuclease was used to cut the target site in exons 1 and 6 in mouse *Prex2* which code for E22 and N204 in the catalytic DH domain, and repair templates (partial sequences shown) were used to introduce the E22A and N204A mutations (red) and neighbouring silent mutations (green). The sites were

targeted sequentially. Further information on the generation of the *Prex2*^{GD} mouse strain can be found in our recent publication on the role of P-Rex2 in melanoma [1]. **(B)** P-Rex2 western blot. Total cerebellar lysates were prepared from 15-week-old male *Prex2*$^{+/+}$ (blue, circles), *Prex2*$^{-/-}$ (purple, squares), and *Prex2*^{GD} (green, triangles) mice. *Prex2*$^{-/-}$ mice were described previously; they seem healthy except for a motor coordination defect that becomes apparent when they are challenged, e.g. on a rotarod [2]. *Prex2*^{GD} mice were as in (A). The cerebellar lysates were western blotted with P-Rex2 antibody. A representative blot is shown. Coomassie staining was used as a loading control. Blots were quantified by Fiji densitometry. Data are mean ± SEM of 3 independent experiments; symbol colours mark individual experiments. Statistics are one-way ANOVA with Tukey's multiple comparisons corrections; p-values in black denote significant differences, p-values in grey are non-significant. **(C, D)** Fasting blood glucose and body weights of *Prex2*$^{-/-}$ and *Prex2*^{GD} mice ageing on high-fat diet (HFD), for comparison with Figure 1B (*Prex2*$^{-/-}$ and *Prex2*^{GD} mice on chow diet). The fasting blood glucose and body weights of 6-month-old male (C) and female (D) *Prex2*$^{+/+}$, *Prex2*$^{-/-}$, and *Prex2*^{GD} mice on HFD, the same mice as in Figures 1C and 1D, were measured. For *Prex2*$^{+/+}$ and *Prex2*$^{-/-}$ males, one additional cohort of 5 mice/genotype was included. Data are mean ± SEM of 12-17 mice per group pooled from 3-4 independent cohorts for each sex. Statistics in time courses are two-way ANOVA with Sidak's multiple comparisons correction; purple stars denote significance between *Prex2*$^{+/+}$ and *Prex2*$^{-/-}$, green between *Prex2*$^{+/+}$ and *Prex2*^{GD}.

[1] Ford, C. A. *et al*. Targeting the PREX2/RAC1/PI3Kβ signaling axis confers sensitivity to clinically relevant therapeutic approaches in melanoma. *Cancer Res* **85**, 808-824. doi: 10.1158/0008-5472.CAN-23-2814 (2025).

[2] Donald, S. *et al.* P-Rex2 regulates Purkinje cell dendrite morphology and motor coordination. *Proc Natl Acad Sci U S A* **105**, 4483-4488, doi:10.1073/pnas.0712324105 (2008).

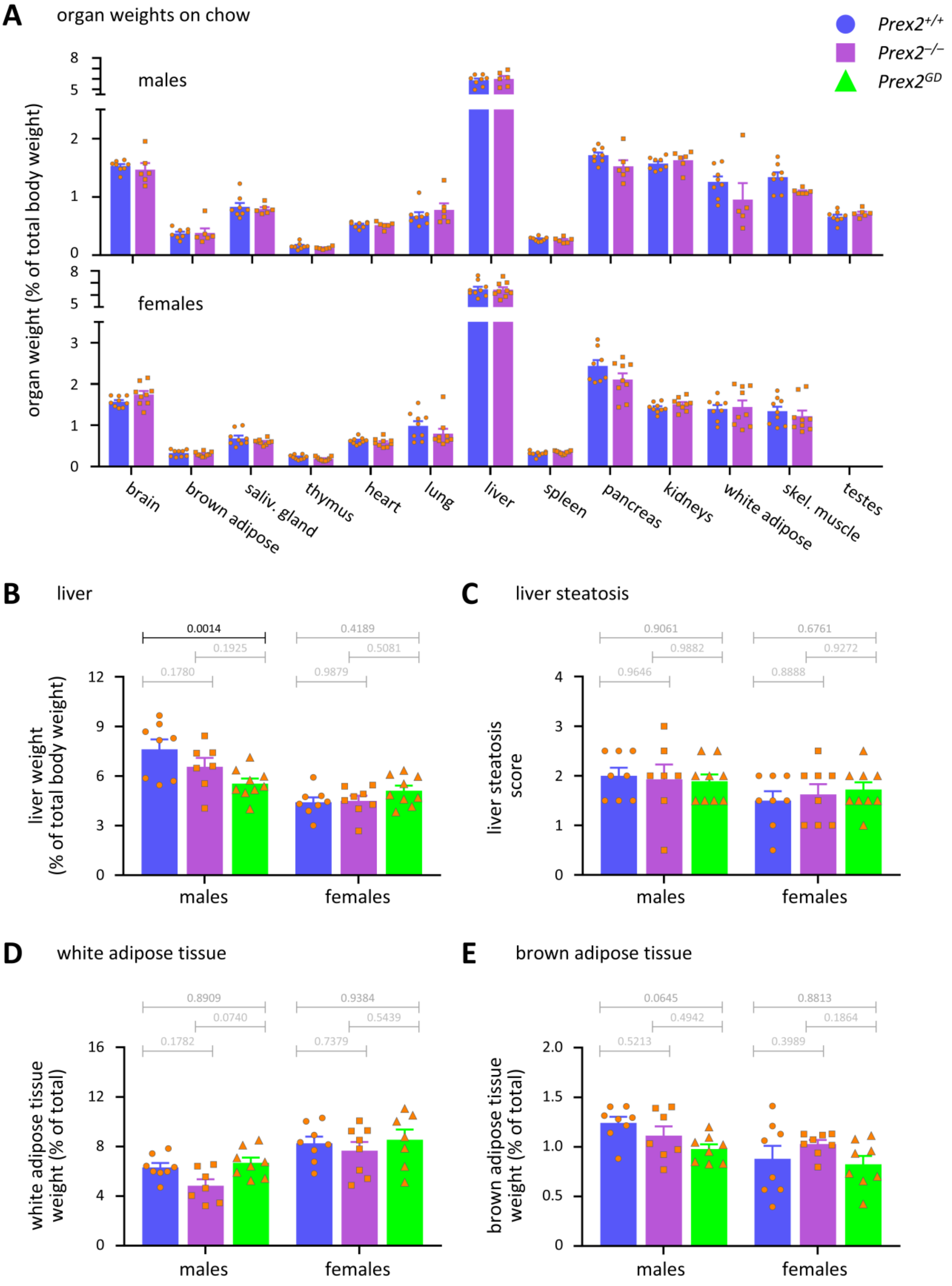

Supplementary Figure 2

Supplementary Figure 2. Organ weights are normal in *Prex2*$^{-/-}$ and *Prex2*^{GD} mice, except for reduced liver size in *Prex2*^{GD} males on HFD. (A) Organ weights on chow diet. 24-week-old male and 30-week-old female *Prex2*$^{+/+}$ (blue, circles) and *Prex2*$^{-/-}$ (purple, squares) mice on chow diet were dissected and their organ weights recorded. Data are mean ± SEM of 6-9 mice per sex per genotype. **(B-E)** Organ weights and liver steatosis on HFD. 52-week-old male and female *Prex2*$^{+/+}$, *Prex2*$^{-/-}$, and *Prex2*^{GD} (green triangles) mice on HFD were dissected and the weights of their (A) liver, (B) white adipose tissue, and (D) brown adipose tissue recorded, and (D) the steatosis level of the livers scored. Data are mean ± SEM pooled of 7-9 mice per genotype from the same cohorts as shown in Figure 1C-D; beige dots show individual mice. Statistics in (A-E) are two-way ANOVA with Sidak's multiple comparisons correction; p-values in black denote significant differences, p-values in grey are non-significant.

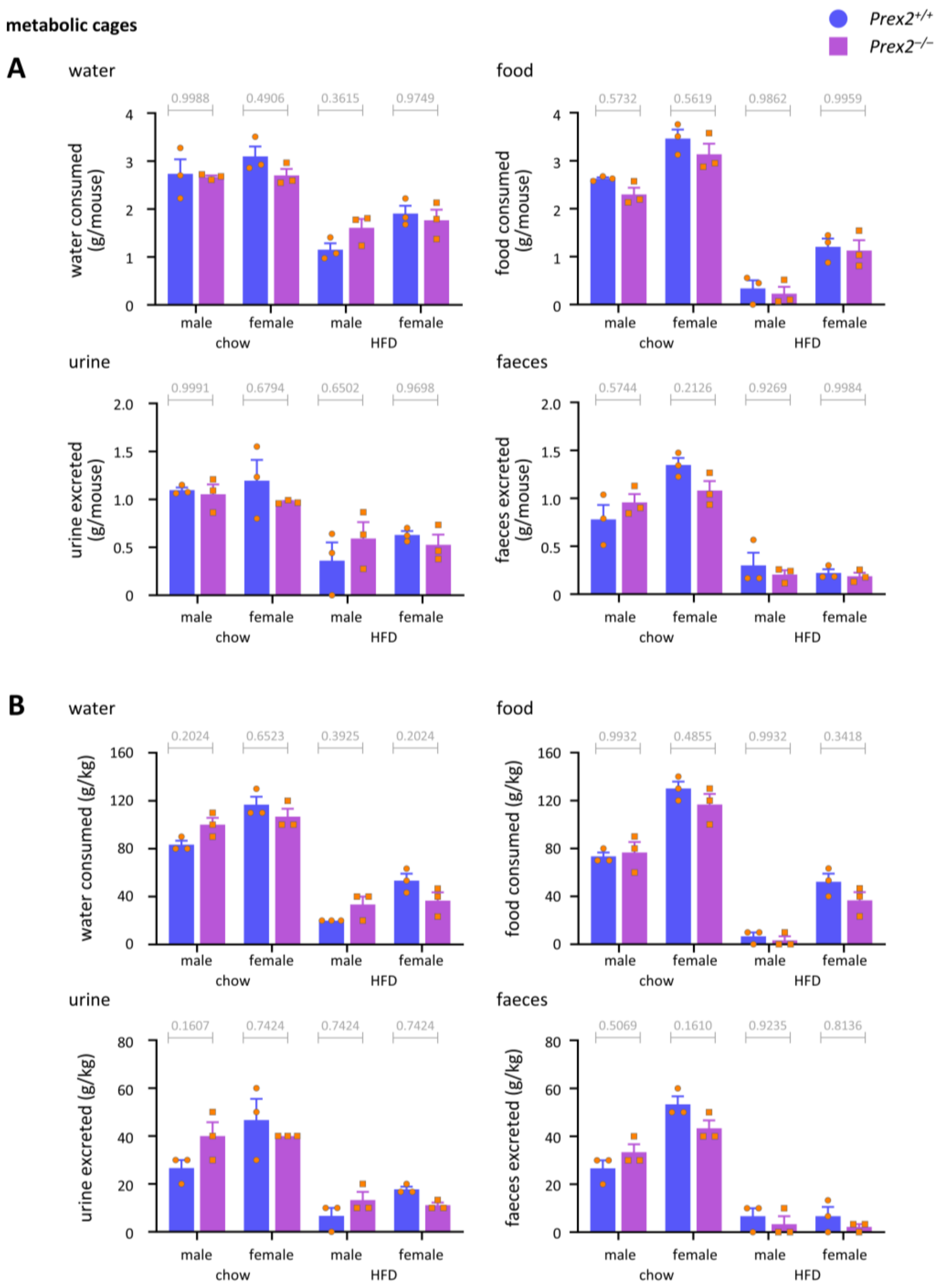

Supplementary Figure 3. *Prex2$^{-/-}$* mice perform normally in metabolic cages. (A, B) 11-months-old male and female *Prex2$^{+/+}$* (blue, circles) and *Prex2$^{-/-}$* (purple, squares) mice on chow diet or HFD, as indicated, were evaluated in metabolic cages (up to 3 mice/cage) on 3 subsequent nights, with food and water *ad libitum*. Water and food consumption, and the production of urine and faeces were measured after each night. Data are mean ± SEM of each night, from 1-3 independent cohorts of 3-5 mice/group. Data in (A) and (B) are from the same mice, in (A) expressed as g/mouse, in (B) as g/kg body weight. Statistics are two-way ANOVA with Sidak's multiple comparisons correction; p-values in black denote significant differences, p-values in grey are non-significant.

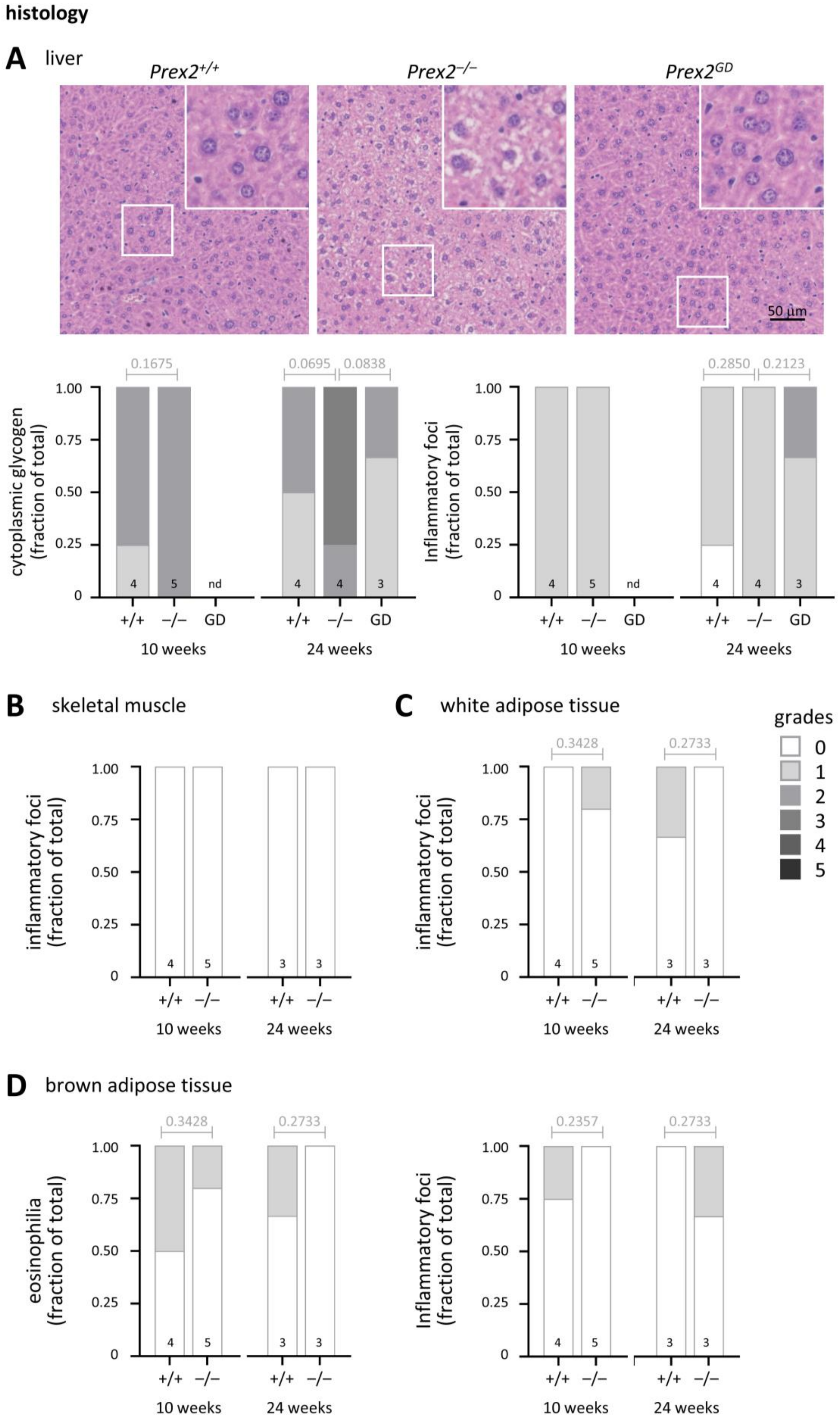

Supplementary Figure 4

Supplementary Figure 4. ***Prex2*$^{-/-}$ mice have a tendency towards increased liver glycogen storage. (A-D)** Histology of metabolic organs. The (A) liver, (B) skeletal muscle, (C) white adipose tissue, and (D) brown adipose tissue of 10-week-old and 24-week-old male *Prex2*$^{+/+}$, *Prex2*$^{-/-}$, and *Prex2*^{GD} mice on chow diet were fixed, sectioned, and H&E stained. The presence of inflammatory cell foci, eosinophil infiltration, and liver glycogen storage were assessed by a pathologist from 0 (normal) to 5 (whole tissue affected). (A) includes representative images of livers from 24-week-old mice (cytoplasmic glycogen appears as white spaces) taken on a Zeiss Axioscan 7 with the 20× objective. Data are mean ± SEM of 3-5 mice/group as indicated in the graphs. Statistics are chi-square test; p-values in grey are non-significant.

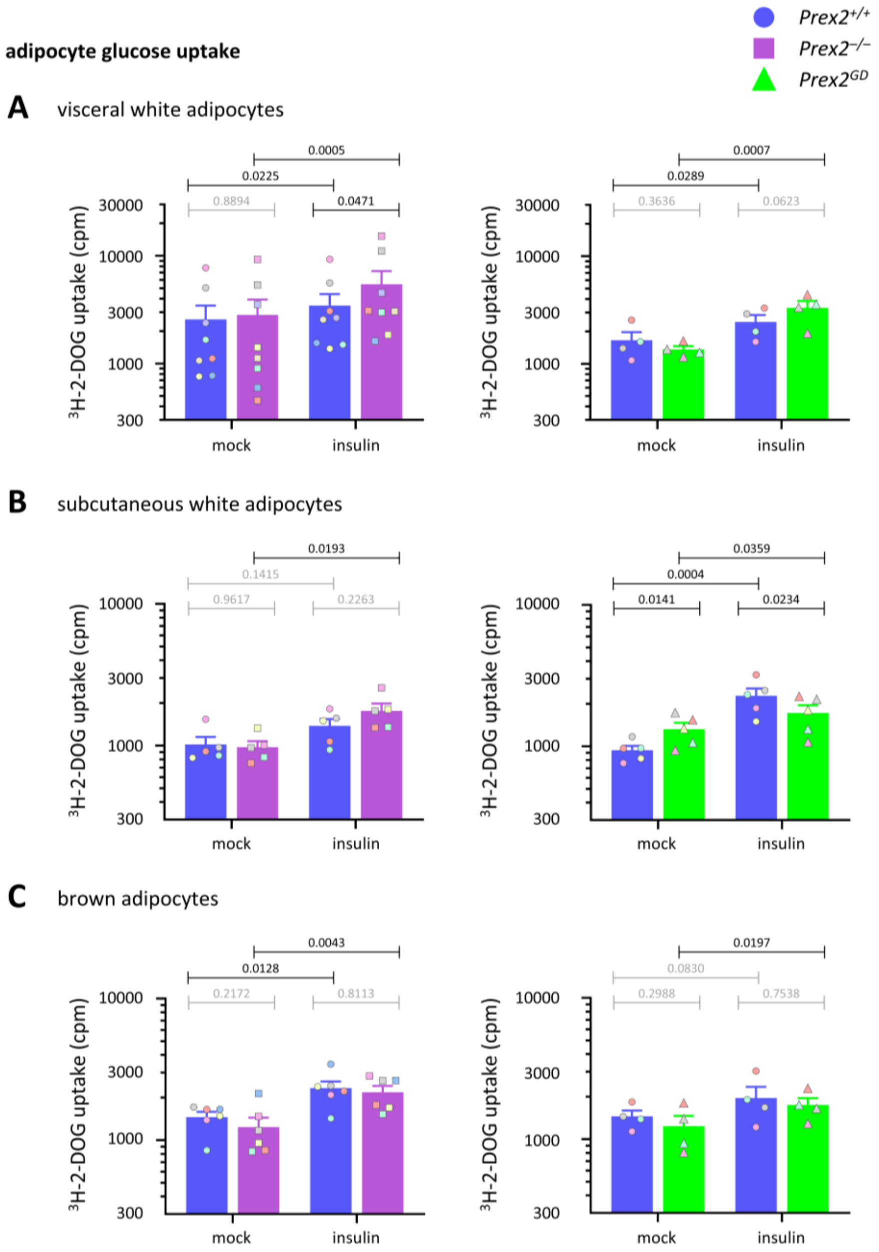

Supplementary Figure 5. P-Rex2 does not control glucose uptake into adipose cells. Glucose uptake was measured in mature **(A)** visceral white, **(B)** subcutaneous white, and **(C)** brown adipose cells isolated from 15-week-old *Prex2*$^{+/+}$ (blue, circles), *Prex2*$^{-/-}$ (purple, squares), and *Prex2*^{GD} (green, triangles) mice on chow diet. Cells were stimulated with 100 nM insulin for 10 min at 37°C, or mock-stimulated, followed by the addition of 50 µM 2-DOG, 0.25 µCi ^{3}H-labelled 2-DOG for 60 min. Cells were washed and glucose uptake measured by scintillation counting. Data are mean ± SEM of 4-8 independent experiments; symbol colours mark individual experiments. Statistics are two-way ANOVA with Sidak's multiple comparisons correction; p-values in black denote significant differences, p-values in grey are non-significant.

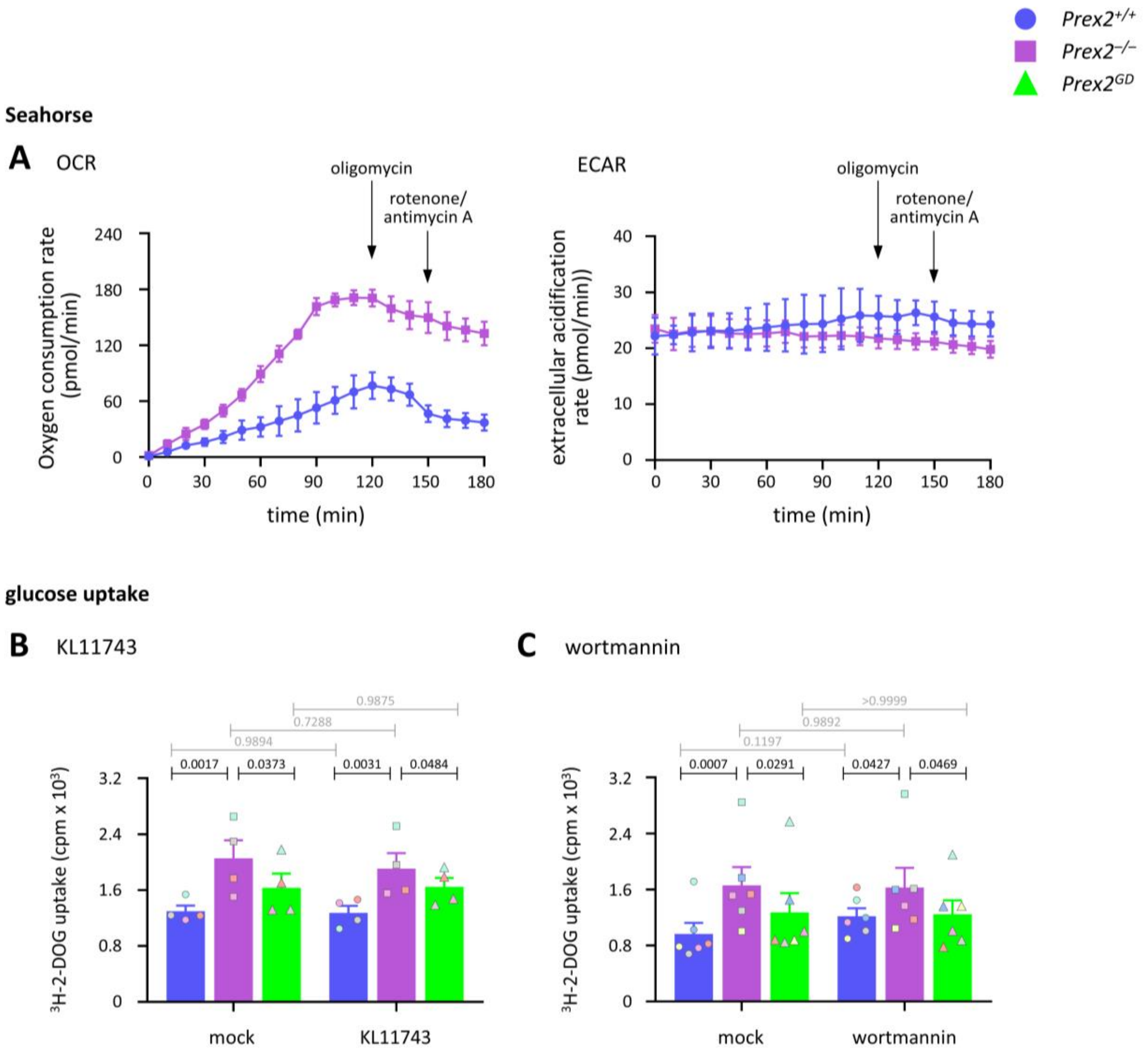

Supplementary Figure 6. P-Rex2 controls mitochondrial respiration and glucose uptake in liver. (A) Liver cells from 15-week-old male *Prex2*$^{+/+}$ (blue, circles) and *Prex2*$^{-/-}$ (purple, squares) mice were analysed by Seahorse assay to quantify oxygen consumption rate (OCR) and extracellular acidification rate (ECAR) as the basis for determination of mitochondrial and glycolytic ATP production. The timepoints of addition of oligomycin and rotenone/antinomycin A are indicated. The traces shown are from one experiment representative of the 5 independent experiments shown in Figure 4C. **(B, C)** Liver cells from *Prex2*$^{+/+}$, *Prex2*$^{-/-}$, and *Prex2*^{GD} (green, triangles) mice were treated with (B) 500 nM KL11743 or (C) 50 nM wortmannin for 3.5 h at 37°C, or mock-treated, followed by the addition of 50 µM 2-DOG, 0.25 µCi ^{3}H-2-DOG for 1 h. Cells were washed, lysed, and glucose uptake was measured by scintillation counting. Data in (C, D) are mean ± SEM of the same (C) 4 and (D) 6 independent experiments as those shown in Figure 5D and 5E, respectively; symbol colours mark individual experiments. Statistics are two-way ANOVA with Sidak's multiple comparisons correction. P-values in black denote significant differences, p-values in grey are non-significant.

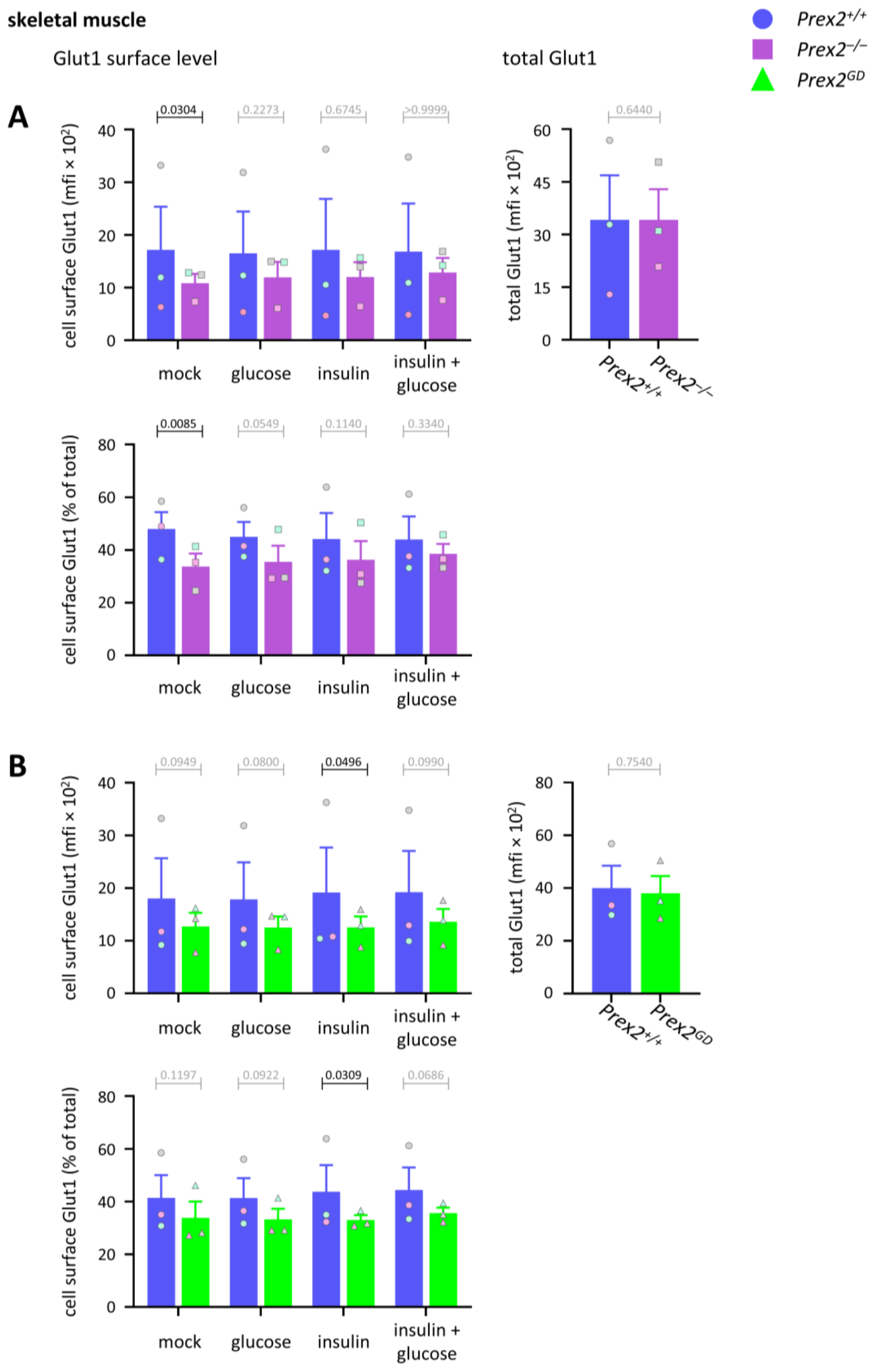

Supplementary Figure 7. P-Rex2 does not affect Glut1 surface level in skeletal muscle cells overall. (A, B) Skeletal muscle cells from 15-week-old male *Prex2*$^{+/+}$ (blue, circles), *Prex2*$^{-/-}$ (A, purple, squares), and *Prex2*^{GD} (B, green, triangles) mice on chow diet were stimulated with 5 mM glucose or 100 nM

insulin for 10 min at 37°C, or mock-stimulated, or were stimulated with 100 nM insulin for 10 min and 5 mM glucose for another 30 min, stained with Glut1 antibody and analysed by flow cytometry. Left: The mean fluorescence intensity (mfi) of Glut1 surface level is expressed as raw mfi values (top) and as % of total Glut1 (bottom). Data are mean ± SEM of 3 independent experiments/genotype; beige symbols show individual experiments. Statistics are two-way ANOVA with Sidak's multiple comparisons correction. Right: Total Glut1 was measured in the same way except in permeabilised cells. Data are mean ± SEM of 3 independent experiments per group; symbol colours mark individual experiments. Statistics are paired t-test. P-values in black denote significant differences, p-values in grey are non-significant.

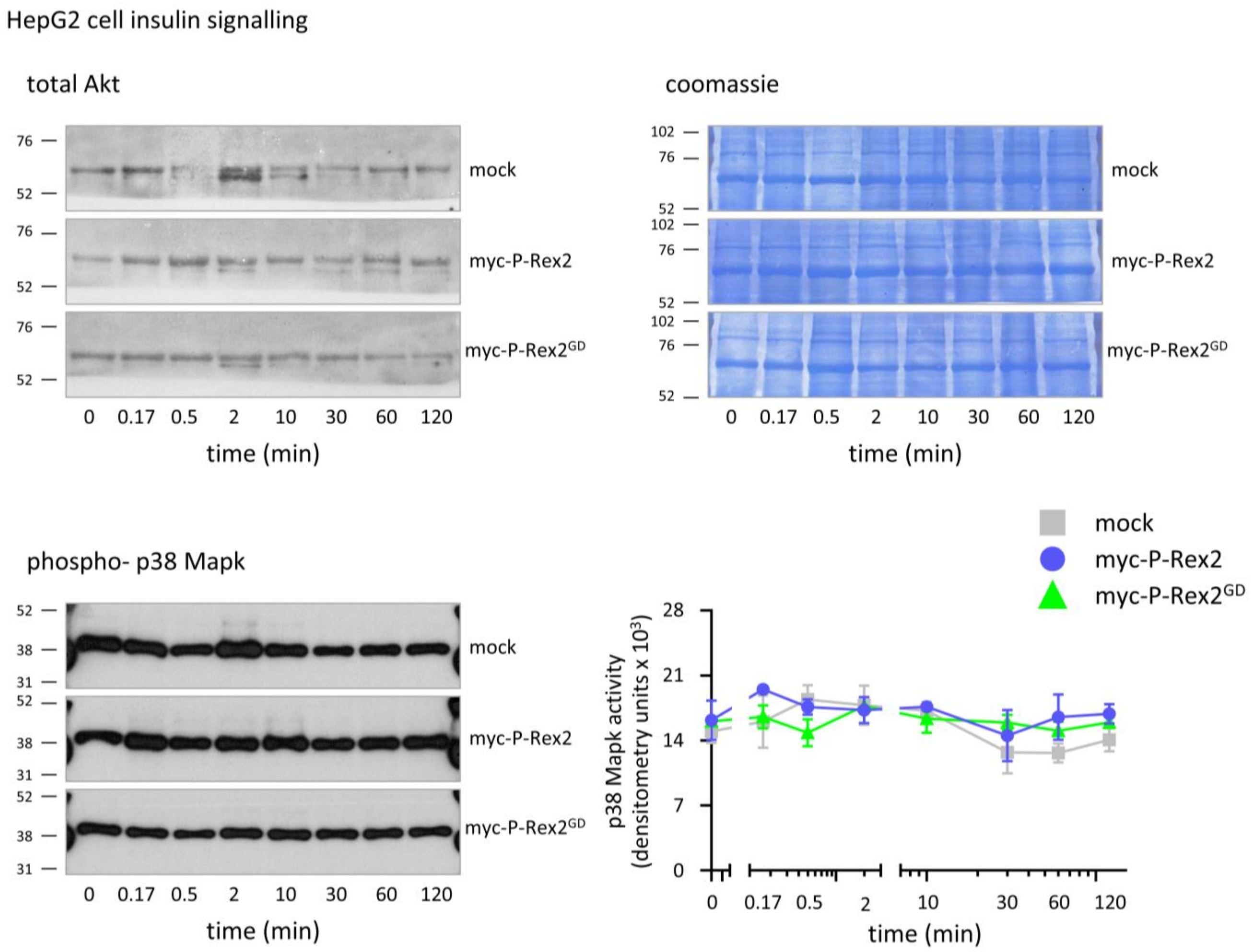

Supplementary Figure 8. P-Rex2 does not control Akt levels or p38 Map activity in HepG2 cells. HepG2 cells were transfected with wild type or GEF-dead pCMV3-myc-P-Rex2, or mock-transfected with empty vector, as indicated, serum-starved, and stimulated with 25 nM insulin for the times shown. Total lysates were western blotted. The top half of the membranes were blotted for phospho-S473 Akt as shown in Figure 7A, then stripped and reprobed for total Akt. The bottom half was blotted for phospho-p38 Mapk and quantified by Fiji densitometry. Representative blots are shown. p38 Mapk activity data are mean ± SEM of 4 independent experiments; statistics (two-way ANOVA with Sidak's multiple comparisons correction) showed no difference between genotypes). Coomassie staining is shown as a loading control.

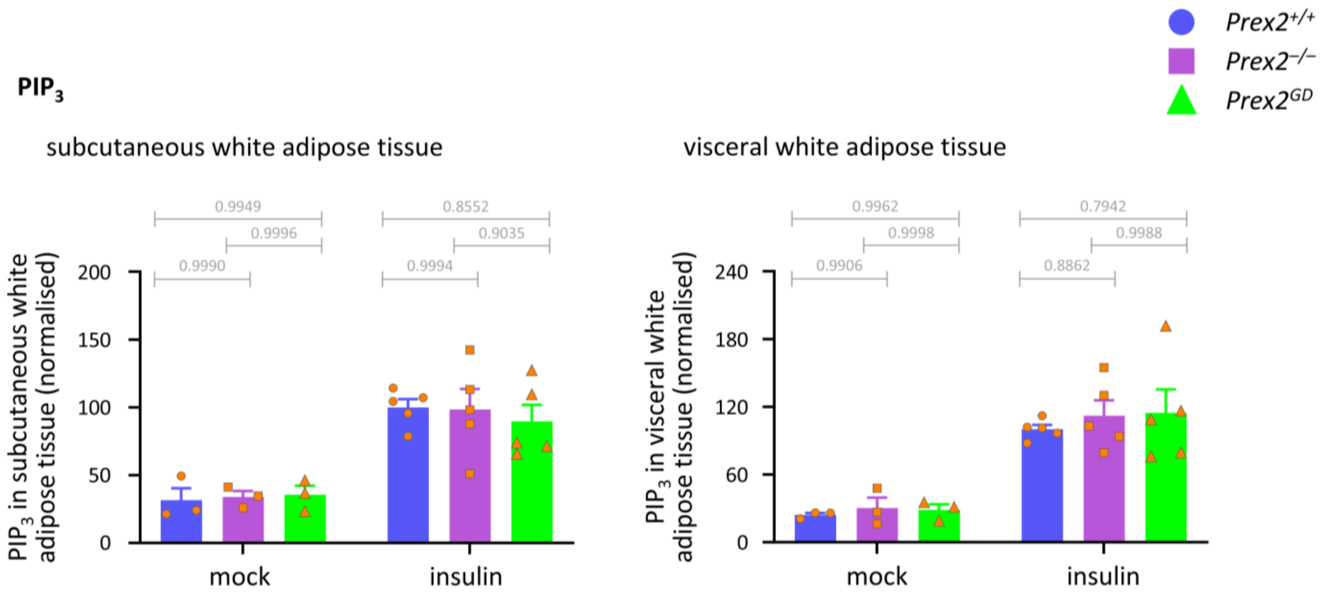

Supplementary Figure 9. P-Rex2 does not affect insulin-stimulated PIP_3 production in adipose tissue. 15-19-week-old male *Prex2*$^{+/+}$ (blue, circles), *Prex2*$^{-/-}$ (purple, squares), and *Prex2*^{GD} (green, triangles) mice on HFD were fasted for 4 h and injected *i.p.* with 10 U/kg insulin or mock-treated. After 8 min, mice were humanely killed, and subcutaneous and visceral adipose tissues were recovered. Lipids were extracted and analysed by HPLC-MS. For each cohort, PIP_3 levels (PIP_3/PIP_2 ratio) were normalised to the mean *Prex2*$^{+/+}$ insulin response. Data are mean ± SEM of 3 mock-stimulated and 5 insulin-stimulated mice/group pooled from two independent cohorts, the same animals as in Figure 7B. Statistics are two-way ANOVA with Sidak's multiple comparisons correction; p-values in black denote significant differences, p-values in grey are non-significant.

Uncropped blots

for Figure 7A

Akt phospho-S473

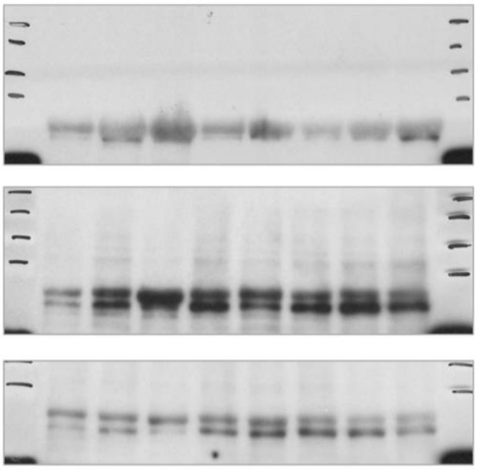

myc

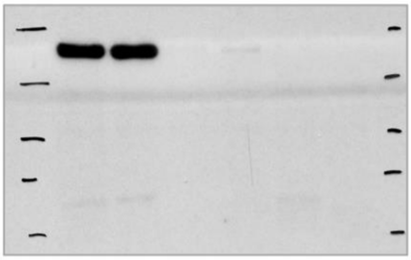

for Supplemental Figure 1B

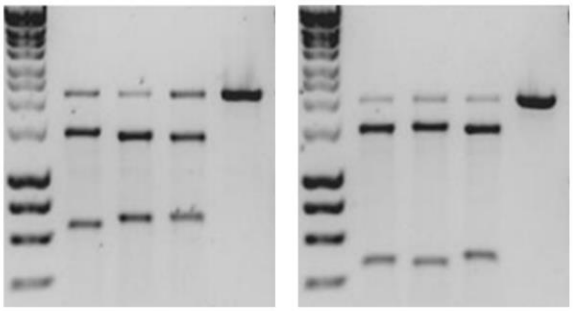

for Supplemental Figure 1C

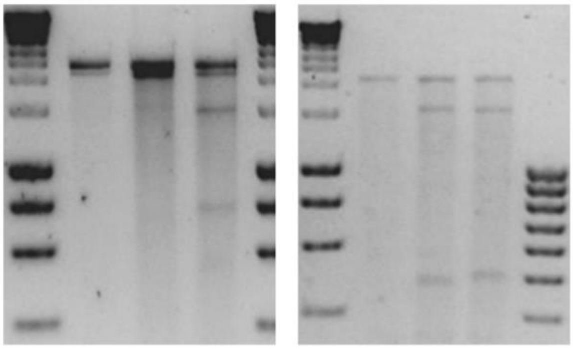

for Supplemental Figure 1F

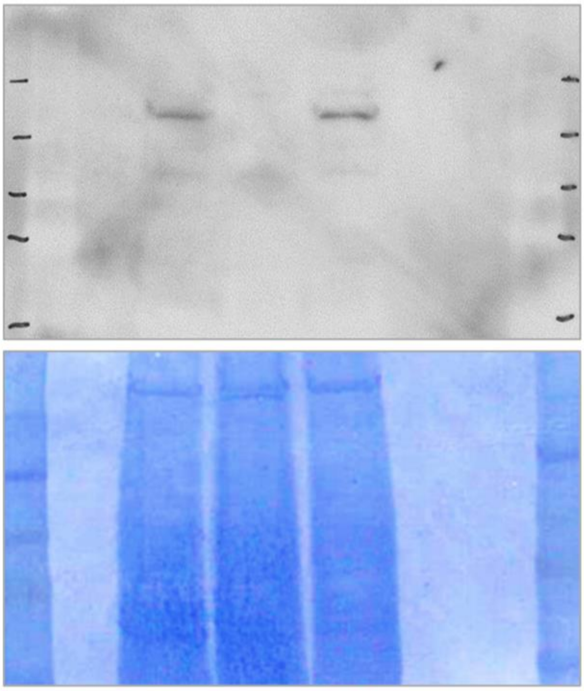

Uncropped blots cont.

for Supplemental Figure 8

total Akt

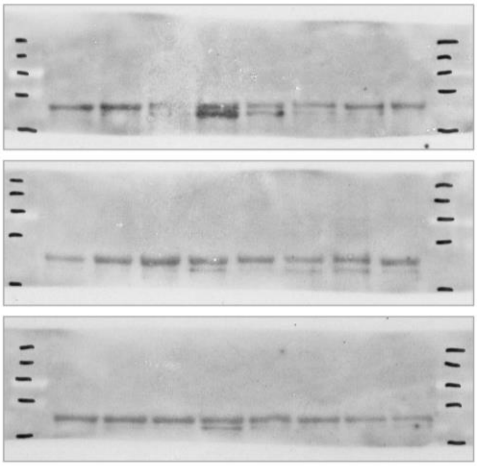

phospho- p38 Mapk

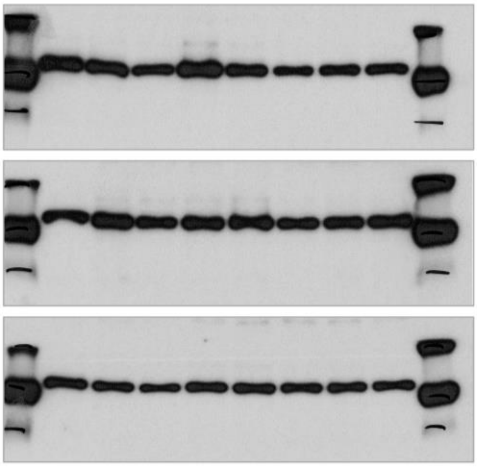

coomassie

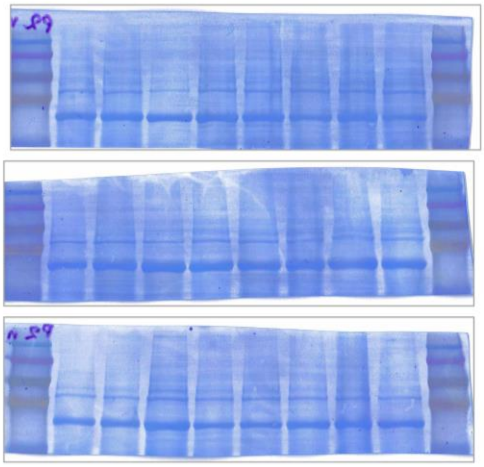

Supplement: Supplementary file 1 — Supplementary Information. [file 41598_2025_1720_MOESM1_ESM.pdf]
